# Generating models for isoform-specific PKM-KIBRA interactions with BIFC, stabilization and AlphaFold 3

**DOI:** 10.1186/s13041-026-01321-2

**Published:** 2026-06-19

**Authors:** Edna Amoah, Tyler W. Dunn, Larissa Ferguson, Kristel Betriu Diaz, Connor O’Donnell, Joachim Kremerskoten, Wayne S. Sossin

**Affiliations:** 1 Department of Neurology and Neurosurgery, Montreal Neurological Institute, McGill University, Montreal, QC H3A 2B4 Canada; 2 Department Molecular Nephrology, Internal Medicine D, University Hospital of Muenster, 48149 Muenster, Germany

**Keywords:** Persistent protein kinases, Synaptic plasticity, Memory retention, Protein complexes, Atypical protein kinase C, PKC

## Abstract

The truncated constitutive active form of protein kinase C (PKC) called protein kinase M (PKM) plays a role in long-term memory maintenance in vertebrate and invertebrate models. Previously we have shown that the *Aplysia* Kidney/Brain protein (KIBRA) stabilizes the atypical PKM Apl III, but not the classical PKM Apl I in *Aplysia* neurons. Expression of *Aplysia* KIBRA with changes in the proposed atypical PKM binding site does not stabilize PKM Apl III and erases forms of plasticity supported by PKM Apl III. Here, we have examined biomolecular fluorescence complementation (BIFC) between KIBRA variants and PKM Apl III in *Aplysia* neurons. These KIBRA variants include: the KIBRA with changes in the proposed atypical binding site noted above; a splicing variant that stabilizes PKM Apl I, but not PKM Apl III; and several mutations identified in mammalian WW and C2 domain containing protein 3 (WWC3, a member of the chordate-specific expansion of the KIBRA family) associated with cancer or neurodevelopmental disorders. Surprisingly, we find that some KIBRA variants show BIFC with PKM Apl III but do not stabilize PKM Apl III. We used models of protein-protein interactions (AlphaFold 3) to gain insights into the discrepancy between BIFC and stabilization of PKMs by KIBRA and KIBRA variants and suggest a model where stabilization is linked to stable inhibition of PKMs by KIBRA.

**Supplementary Information:**

The online version contains supplementary material available at 10.1186/s13041-026-01321-2.

## Introduction

A major challenge in our understanding of changes in synaptic strength underlying memory is how these changes are maintained for long periods of time, but at the same time can be erased by pharmacological agents. We have proposed that this challenge can be overcome by specialized synapses where synaptic strength is maintained by persistent protein kinases underlying memory maintenance [39]. We have examined this hypothesis in *Aplysia* sensory-motor neuron synapses, where one can study long-term changes in synaptic strength related to long-term memory in a reduced system [21]. In these cultures, increases in synaptic strength associated with memory can be induced and maintained for days [17]. The increase in synaptic strength (and memory) in *Aplysia* are erased by inhibitors that target persistent protein kinases, in particular truncated forms of PKC that are termed PKMs [4, 15]. These inhibitors erase memory in many systems consistent with a model where mechanisms underlying long-term changes in synaptic strength, but not short-term changes, are highly conserved over evolution [52].

Two different forms of long-term facilitation, LTF can be induced in *Aplysia* sensory-motor neuron cultures, non-associative LTF (implicated in sensitization memory) induced by repeated applications of serotonin, and associative LTF (implicated in associative memory), induced by coupling firing of the sensory neuron and serotonin [15]. PKMs are formed by calpain cleavage in *Aplysia* and PKMs formed from classical PKCs (PKM Apl I) and atypical PKCs (PKM Apl III) play distinct roles in synaptic plasticity underlying memory formation [3, [8], 15] . Dominant negative forms of PKM can erase LTF, even if expressed two days after the increase in synaptic strength was induced, but distinct dominant negative PKMs block associative and non-associative LTF [15, 16]. Vertebrate PKMζ binds to the adaptor protein KIBRA and altering three residues in the proposed PKM binding domain block this association [45]. Expression of *Aplysia* KIBRA with these three conserved residues changed (arginine 1317,serine 1319, arginine 1321 converted to alanines, KIBRA_RSR−AAA_), erase LTF when injected into the sensory or motor neurons, matching the effects of dominant negative atypical PKM Apl III [16]. Drugs and peptides that interfere with KIBRA-PKMζ association also reverse synaptic plasticity and memory in vertebrate models [44].

KIBRA stabilizes PKMζ in vertebrates. Mice without KIBRA have significantly lower levels of PKMζ, consistent with an endogenous role for KIBRA in stabilizing PKMζ [45]. It is not clear if stabilization is simply due to association of PKMs with KIBRA. For example, KIBRA stabilizes the kinase LATS, but this is largely independent of its binding to LATS through its WW domains [49]. In contrast, KIBRA stabilization of PKMζ in vertebrates has been linked to its association since the alteration mentioned above, KIBRA_RSR−AAA_, shows neither stabilization nor co-immunoprecipitation [22, 45]. KIBRA also stabilized PKM Apl III in Aplysia sensory neurons but did not stabilize PKM Apl I [16]. Similar to vertebrates, KIBRA_RSR−AAA_ does not stabilize PKM Apl III in *Aplysia* neurons but surprisingly does stabilize PKM Apl I [10, 16]. There is an alternatively spliced insert in *Aplysia* KIBRA [10]. Like KIBRA_RSR−AAA_, KIBRA_splice_ stabilizes PKM Apl I, but not PKM Apl III [10]. PKM Apl I is required in the postsynaptic neuron for maintenance of non-associative LTF [15].

KIBRA is a conserved adaptor protein implicated in cell polarity, the Hippo pathway and cell proliferation [11, 41, 51]. Atypical PKC is also implicated in these pathways and KIBRA and the atypical PKC are both participants in protein complexes implicated in these pathways [42, 51, 53]. KIBRA polymorphisms in humans are associated with cognitive differences [2, 32, 33] and in neurons, KIBRA, also known as WWC1 and its other family members, WWC2 and WWC3 (formed by duplications during chordate evolution) localize to synapses and regulates both behavior and synaptic physiology underlying memory and behavior [6, 12, 22, 28, 29, 30, 43, 44, 45].

KIBRA variants show differential stabilization of PKM Apl I and PKM Apl III [10]. However, there has been no investigation of the relationship between interaction and stabilization in *Aplysia*. BIFC allows for measurements of interaction in a cellular environment [25, 26] and has been used to examine KIBRA-PKMζ interactions in vertebrate cells [44, 45]. Here, we determine, for PKM Apl III, if changes in stabilization are correlated with changes in BIFC. Mutations in WWC3 near to the proposed PKM binding site are linked to neurodevelopmental disorders or cancer [18, 19, 40]. We also examined the effects of these mutations on stabilization of PKM Apl I and PKM Apl III and BIFC with PKM Apl III. Overall, we found that association measured by BIFC is not always correlated with stabilization. To gain insight into this, we have used AlphaFold 3 to model the KIBRA-PKM interaction and results from this model suggest that stabilization is linked to interactions where KIBRA stably inhibits PKM.

## Materials and methods

### Aplysia cell culture

Hermaphrodite *Aplysia californica* from Miami *Aplysia* Resource Facility (RSMAS) were anesthetized via injection of 50–60 mL of 400 mM isotonic MgCl_2_ and pleuropedal ganglia were removed. All animal experiments were carried out in compliance with protocols approved by the Montreal Neurologic Institute animal committee (Protocol MNI-5784). Ganglia were digested at 19 °C in L15 media containing 10 mg/ml Dispase II (Roche Diagnostics) for 18–19 h. L15 medium (Sigma-Aldrich) was supplemented with 0.2 M NaCl, 26 mM MgSO_4_, 35 mM dextrose, 27 mM MgCl_2_, 4.7 mM KCl, 2 mM NaHCO_3_, 9.7 mM CaCl_2_, and 15 mM HEPES, with pH 7.4. Glass bottom culture dishes were coated with 0.05% poly-L-lysine for 1–2 h and washed with ddH_2_O prior to use. Sensory neurons were isolated from pleural ganglia that were dissected from adult *Aplysia* (60–100 g). Neurons were cultured in 50% hemolymph/50% L15 media supplemented with L-glutamine. All experiments were performed at 19 °C.

### Plasmid constructs and microinjection

All constructs were made in the pNEX3 vector [20]. mRFP-PKM Apl I, mRFP-PKM Apl III, KIBRA_WT_, KIBRA_RSR−AAA_ and KIBRA_splice_ were previously described [10]. The enhanced cyan fluorescent protein (eCFP)-KIBRA fusion proteins were made using PCR to amplify eCFP with BspE1 and KpN sites and inserted into the pNEX-KIBRA_WT_, KIBRA_RSR−AAA_ or pNEX-KIBRA_splice_ plasmid cut with the same sites. Three GBLOCKS (Integrated DNA technology, IO, USA) were purchased with: the arginine at residue 1312 converted to glutamine; KIBRA_R−Q_; the arginine at residue 1341 converted to histidine KIBRA_R−H_; and the arginine at residue 1312 converted to a stop codon KIBRA_stop_). These GBLOCKs were cut with Afe I and AflII and inserted into the CFP-KIBRA_WT_ or CFP KIBRA_splice_ cut with Afe I (partial) and Afl II. GBLOCKs for the long N-terminal part of mVENUS (1-154) and short C-terminal part of mVENUS (155–239) [24] were generated with restrictions enzymes Sbf1 and PpuM1 and inserted at these sites in all pNEX-KIBRA variants or with restrictions enzymes Age I and Xho I to be inserted into pNEX-mRFP PKM Apl III. The YFP fragments were separated from PKM or KIBRA with a GGGGSGGGGS linker. All plasmids were confirmed by sequencing. For stabilization experiments, molar equivalent levels of plasmids encoding KIBRA variants and mRFP-PKMs were used. A solution containing the desired constructs (max 0.4 ug/ul of DNA in ultrapure water) with 0.2% fast green were microinjected into the nuclei of neurons using back-filled glass micropipettes. A short pressure pulse was delivered to release plasmid solution into nucleus. Neurons were incubated at 19 °C for 24 h to allow sufficient time for expression of injected plasmids prior to imaging.

### Stabilization experiments

To measure stabilization by KIBRA, we express mRFP-tagged PKMs in the sensory neuron in the presence or absence of eCFP-KIBRA variants and measure the intensity of mRFP in processes (to avoid pigment granules in the cells soma that interfere with the measurement) normalized to the expression of eYFP (the plasmid encoding eYFP is co-injected with plasmid expressing mRFP-tagged PKMs) [10]. A blind observer chooses one region of interest (ROI) per neuron representing a sensory neuron neurite of intermediate thickness for the measurement. While some previous experiments used multiple ROIs, variability between different ROIs is low [5] and previous stabilization experiments used one ROI [10]. The level of expression is measured at 24 h after injection and low levels of plasmids are used to minimize toxicity [10].

Images were captured by an LSM 710 (Zeiss) laser confocal scanning microscope equipped with an Axiovert 100 inverted microscope (Zeiss) and a 40x, NA 1.4 objective. The eCFP, eYFP and mRFP, images were acquired sequentially. Fluorescence intensity (arbitrary units) was measured by the Microcomputer-Controlled Imaging Device (MCID) software package (Imaging Research). Background fluorescence from a neighboring region was subtracted from the fluorescence values measured.

### BIFC experiments

To measure interaction by BIFC, we expressed three plasmids, two fusion proteins with non-fluorescent protein fragments, one long fragment (mVENUS 1-154) one short (mVENUS 155–239), and one encoding eCFP as a marker of how much plasmid is injected in sensory neurons. Fluorescence excitation and emission were achieved with an Xcite Expo arc, which delivers rich spectral excitation and uniform widefield fluorescence illumination to the sample plane. The images were captured through epifluorescence microscopy (widefield), and eCFP, eYFP and in some cases red images were acquired sequentially. Sensory neurons were imaged with a Zeiss Axio Observer D1, an EC Plan Neofluar 1.31 Numerical aperture 40× oil coupled lens, and a QuantEM: 512SCEM-CCD camera (Photometrics). The glass-bottom dishes were filled with 1X PBS instead of fresh L-15-based media to avoid background fluorescence. Rapid image acquisition was achieved with a Zeiss SVB-1 microscope signal distribution box and Axiovision 4.8 software using the fast acquisition application (Zeiss). Initiation of the Axiovision image acquisition triggers the recording of three channels (DIC, eCFP, eYFP) or in addition Red (for AlexaFluor647 used in antibody staining). Similar, to stabilization experiments, A blind observer chooses one ROI per neuron representing a sensory neuron neurite of intermediate thickness for the measurement. We measured the intensity of eYFP in processes and normalized it to the intensity of eCFP. For expression levels we measured the intensity of Red staining and normalized it to the intensity of eCFP. For all measurements, a region next to the neurite was chosen for background subtraction.

In some experiments where an antibody to KIBRA was used, cultured cells were fixed with 4% PFA + 30% sucrose in PBS for 30 min and washed with PBS. Fixed cells were permeabilized with 0.1% Triton X-100 with 30% sucrose in PBS for 10 min, and free aldehydes were quenched (50mM ammonium chloride for 10 min, followed by four washes. Cells were incubated with 10% normal goat serum (Sigma Aldrich) and 0.5% Triton X-100 in PBS for 30 min to block nonspecific antibody binding. Cells were then treated with KIBRA Antibody [16] in blocking solution (1:500) for 1 h, followed by five PBS washes (10 min each). Cells were then incubated with AlexaFluor-647-conjugated donkey anti-rabbit antibody in blocking solution (1:500; Invitrogen, REF A315773) for 24 h a 4 °C in darkness and washed with PBS as described above. Imaging was the same as above.

### Quantification and statistics

In all experiments, N represents one sensory neuron. An independent experiment is defined when sensory neurons are obtained at the same time from the same animals and conditions are done in parallel. Sensory neurons from multiple animals are pooled before plating for each independent experiment.

Statistical methods were not used to predetermine sample size. For stabilization experiments, previous experiments [10, 16] indicated that a minimum of three independent experiments and an N of greater than 15 sensory neurons was sufficient to show an effect of KIBRA on stabilization. Since there were multiple constructs and not all constructs were included in each experiment, the number of experiments and sensory neurons were always greater than this minimum. For BIFC experiments, we had not established the variability of this measurement and planned 4 independent experiments for each construct, each including positive (KIBRA_WT_ Long/PKM Apl III short) and negative (PKM Apl III long/PKM Apl III short) controls. Similar to stabilization experiments, since there were multiple constructs and not all were included in each experiment, the number of experiments were greater than this minimum.

For each independent experiment, measures (including the control group) were normalized to the average of the control group (pNEX alone in stabilization experiments, KIBRA long/PKM short in BIFC experiment, KIBRA_WT_ in expression studies). There was no normalization for the initial characterization of BIFC constructs. All quantifications were performed blind to the experimental group.

No test for outliers was done. Shapiro-Wilk tests for normality were done for all groups and for all experiments, at least one group did not pass the normality test (*p* < 0.05). Thus, for all experiments, the non-parametric Kruskal-Wallis test was used to determine if there were significant differences between the groups. Pre-planned Dunns Post-Hoc tests were planned to test for significant stabilization or BIFC compared to the negative control (pNEX for stabilization, PKM Apl III long/PKM Apl III short for BIFC). All statistical tests were done with GraphPad Prism version 9.

### AlphaFold3 simulations

Structural models were generated with AlphaFold 3 [1] on the AlphaFold 3 server (https://alphafoldserver.com/). All described interactions are predicted and have not been experimentally validated. The terms ‘interaction,’ ‘binding,’ and ‘contact’ in the context of AlphaFold 3 results refer to predicted structural proximity, not confirmed molecular interactions. These models were generated with default template settings (PDB templates with default cut-off date (29/09/2021). The proteins used were PKM Apl III (NP_01191516.1 starting at M238 with PTMs as pT179 and pT331, PKM Apl I (NP_00191415.1 starting at Y300 preceded by 7 linker resides (SGLRSRG) with PTMs at pT186, pT328, and pS347), PKM zeta (NP_001229803.1 starting at M80 with PTMs at pT227 and pT377), KIBRA_WT_ ( XP_35825059.1), KIBRA_splice_ (XP_035825058.1), Human KIBRA (NO_001155133.1), Human WWC2 (NP_0013977931.1), Human WWC3 (N_056506.3), Par3 (NP_001171720.1). For KIBRA mutants (KIBRA_RSR−AAA_, KIBRA_R−Q_, KIBRA_R−H_ and KIBRA_stop_) all models used the same sequence as KIBRA_WT_ with the changes described in the text. All models also contained ATP and 2 Mg^2+^ ions. Unless otherwise stated, all queries were run five times to generate 25 models, to sample differences that might arise from the multiple sequence alignment process and the random model generation start sites. All models were subsequently analyzed with Molprobity [47]; hydrogens were added to models with Reduce (NOFLIP flag) with electron-cloud x-H bond lengths to preserve predicted side chain orientations, then the models were scanned for residue atomic clash above 0.4Å and Ramachandran probability scores. Mean clash per residue was calculated as the mean significant clash (> 0.4Å) per residue over the stated region from individual residue clash matrices. Representative models were selected as the model with no severe clashes (> 0.9Å), 100% favored Ramachandran scores, and the lowest overall mean clash per residue over the canonical substrate interface (KIBRA_WT_ 1310–1323). AlphaFold generated predicted Local Distance Difference Test (pLDDT) is reported as the mean±pooled SD for the stated region and AlphaFold generated interface predicted Template Modelling **(**ipTM) reported is whole model ipTM. AlphaFold 3 output data from sequence queries were subsequently analysed and visualized with Pymol Molecular Graphics System [36]. For all models with two or more proteins, for each residue the minimum interchain Predicted Alignment Error (interchain PAE or iPAE) was found to identify regions of potential protein-protein interaction. The lower the minimum iPAE (Å) for a residue is, the more confidence in the position of that residue with respect to a residue in the other protein, indicative of participation in a protein-protein interaction. The mean minimum iPAE averages the data over all 25 models to report the relative consistency of the interactions observed in the individual models. The minimum iPAE data highlights focused predicted protein-protein interactions, where the measure directly reflects changes in predicted interaction strength and the specific predicted location of the interaction. However, not all likely protein-protein interactions will be modelled with low iPAE at the interacting residues. If an interaction occurs through a domain that allows alternative residue interactions, iPAE may be relatively low, as specific residues are modeled interacting with a variety of residues in the other protein. To identify such potential protein-protein interactions involving domains we scanned all models with Pymol for interactions, including hydrophobic based on residue identity and < 4Å sidechain proximity, hydrogen bonds based on N/O proximity (< 3.5Å) and donor/acceptor angle (> 120°, yellow dashed lines in models), salt-bridges through residue identity and proximity (< 4.5Å, green dashed lines in models are < 4.0Å), and pi- interactions through residue identity, proximity and angle to ring normal (< 4.5Å; <45° cation-pi, purple dashed lines) that may underlie protein-protein interactions. Domains involved in potential protein-protein interactions show regular interactions with the same target domains in the other protein over the twenty-five models. Error bars on mean minimum iPAE charts are standard error of the mean.

## Results

### KIBRA and PKM Apl III show BIFC, even when they do not show stabilization

**Fig. 1:**
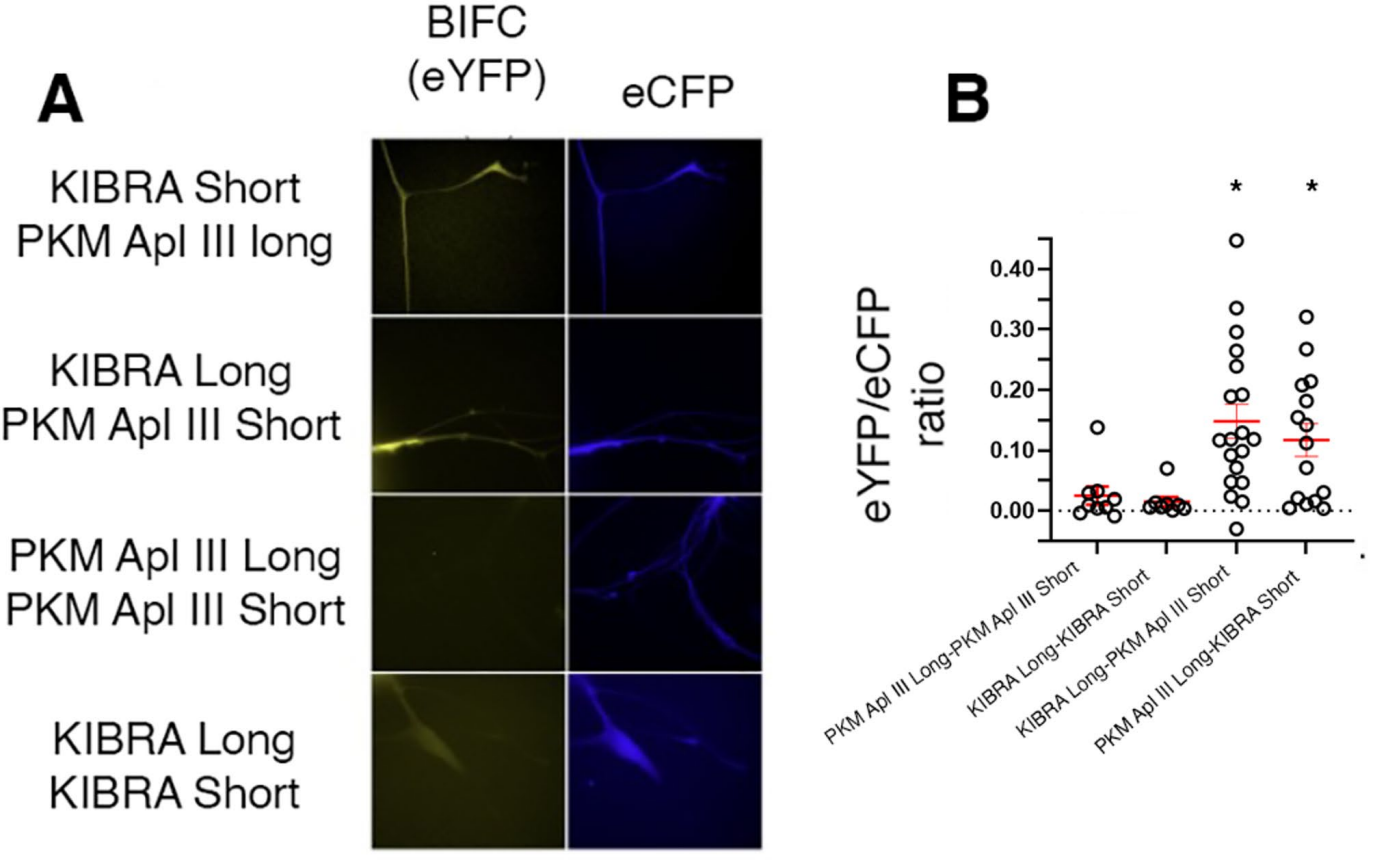
BIFC between KIBRA and PKM Apl III in *Aplysia* sensory neurons. **A** BIFC construct expression where long refers to the N-terminal of YFP (1-154) and short refers to the C-terminal of YFP (155–238). Representative examples of expression of eCFP (control for injection), and eYFP (BiFC) are seen for the four groups (Kibra Short/PKM Long, KIBRA Long/PKM Apl III Short, KIBRA Long/ KIBRA Short, PKM Apl III Long/ PKM Short). **B** All individual points (black circles) and the average and SEM (red) of the unnormalized ratio of eYFP/eCFP is shown. Kruskal-Wallis test (51,4) = 17.52, *p* < 0.001. Dunn’s multiple comparison test showed that PKM Apl III long/PKM Apl III short was significantly different than KIBRA long/PKM Apl III short (*p* < 0.05, *) and PKM Apl III long/Kibra short (*p* < 0.05, *) and KIBRA long/KIBRA shot is significantly different than KIBRA long/PKM short (*p* < 0.01). Results from four independent BIFC experiments with the following number of sensory neurons: PKM Long/PKM Short 9, KIBRA Long/KIBRA Short 8, KIBRA Long/PKM Apl III Short 19, KIBRA Short/PKM Apl III Long 18

In BIFC, association of two proteins, one containing an N-terminal fragment (long, residues 1-154) of eYFP and the other containing a C-terminal fragment (short, residues 155–238) of eYFP allows formation of eYFP when the two proteins associate [23, 24]. We quantified the amount of yellow fluorescence in sensory neuron processes as pigment granules in the cell soma of sensory neurons interfere with measurements of fluorescence in the soma [10]. To control for the level of expression, we co-injected a plasmid encoding eCFP and used the eYFP/eCFP ratio as a measure of BIFC. KIBRA and PKMs were injected with an equal molar ratio of their encoding plasmids. We used similar placement of the two YFP fragments at the N-terminal of KIBRA and PKM as used for BIFC in mammalian cells [44, 45]. We compared the amount of yellow fluorescence to when the two fragments of eYFP were both on KIBRA or both on PKM and found significantly more YFP when the fragments were on KIBRA and PKM, demonstrating an interaction between KIBRA and PKM in sensory neurons that could be monitored by BIFC (Fig. 1).

**Fig. 2:**
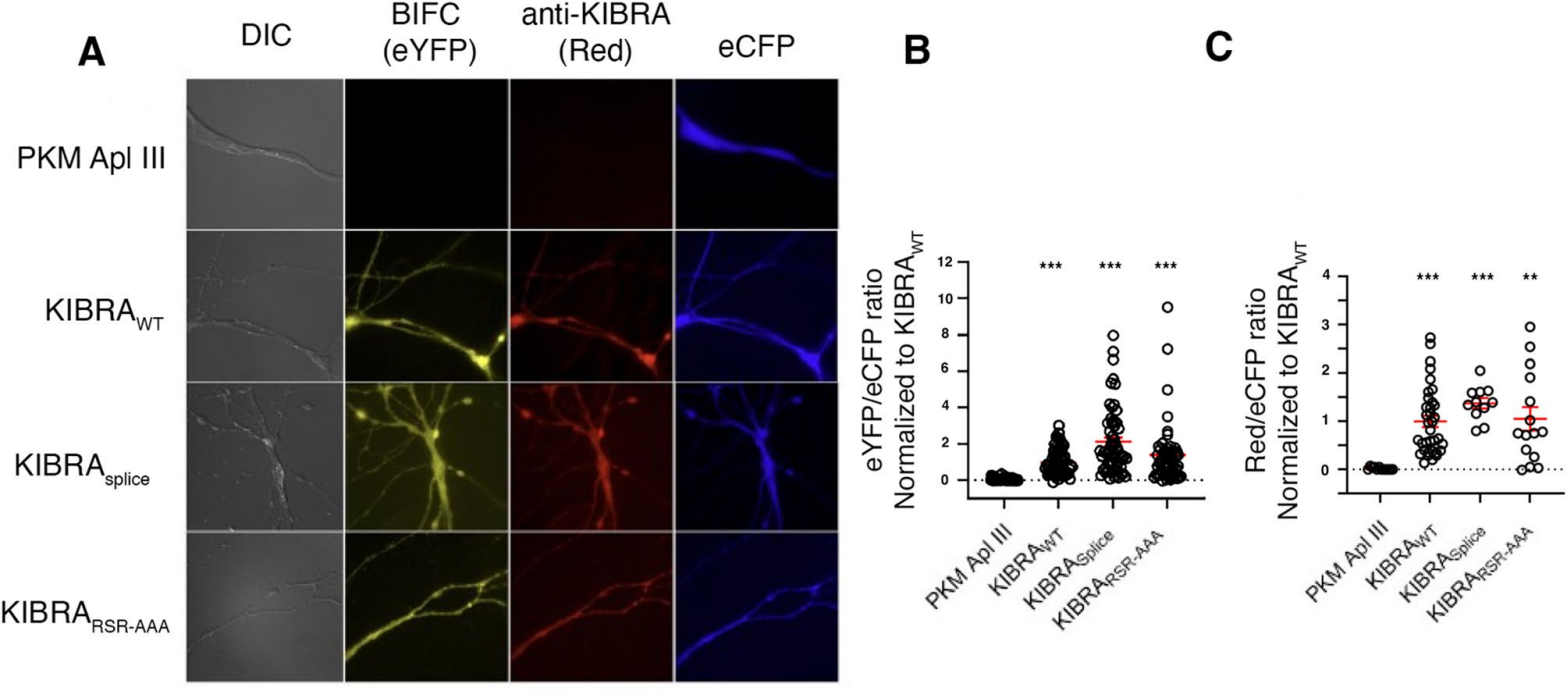
Comparing BIFC between KIBRA variants and PKM Apl III **A** Representative examples of expression of DIC (Differential Interference Contrast), eYFP (representing BIFC signal), red (Kibra Antibody), and eCFP (control for injection) are seen for the four groups all expressing PKM Apl III short and expressing one of PKM Apl III Long, KIBRA_WT_ Long, KIBRA_splice_ Long, KIBRA_RSR−AAA_ Long. **B** All individual points (black circles) and the average and SEM (red) of the normalized eYFP/eCFP ratio (see Methods) is shown. Kruskal-Wallis test (221,4) = 105.9, *p* < 0.0001. Dunn’s multiple comparison test showed that all groups were different than PKM Apl III Long (*p* < 0.001, ***). The N was the number of sensory neurons quantified (PKM Long 46, KIBRA_WT_ Long 64, KIBRA_splice_ Long 59, KIBRA_RSR−AAA_ Long 52) and results are from 8 independent experiments. **C** All individual points (black circles) and the average and SEM (red) of the normalized Red/eCFP ratio is shown. Kruskal Wallis test (73,4) = 30.32, *p* < 0.0001. Dunn’s multiple comparison tests showed that all groups are significantly different than PKM Apl III (only endogenous KIBRA) with *p* < 0.001 (***), or for KIBRA_RSR−AAA_, *p* < 0.01,**). There were no differences between the KIBRA expressing groups (*p* > 0.1). Numbers are different from the BIFC experiments, as antibody staining was not done for all experiments. The N was the number of sensory neurons quantified PKM Apl III Long 12, KIBRA_WT_ 35, KIBRA_RSR−AAA_ 11, KIBRA_splice_ 15) from 4 independent experiments

We examined BIFC for two variants of KIBRA that led to no significant stabilization of PKM Apl III, KIBRA_RSR−AAA_ [16], and KIBRA_splice_ [10], and compared results with KIBRA_WT_. We were surprised to find that in both cases, modified KIBRAs showed strong significant BIFC (Fig. 2). The levels of expressed KIBRA were comparable as monitored by an anti-KIBRA antibody (Fig. 2). Thus, association of KIBRA and PKM measured by BIFC does not always lead to stabilization of PKM.

### AlphaFold 3 modeling of KIBRA-PKM interactions reveals alternative substrate peptides in KIBRA

**Fig. 3:**
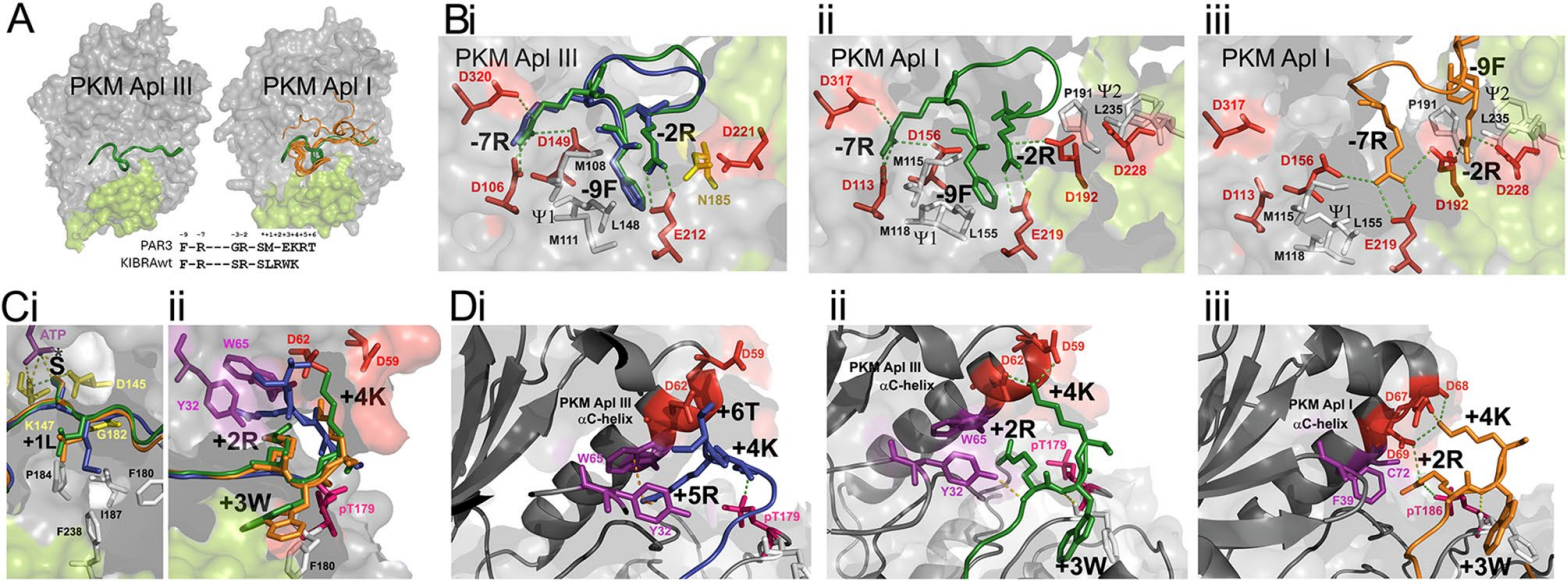
AlphaFold 3 models of Aplysia KIBRA_wt_ or Human PAR3 substrate peptides with PKM Apl III and PKM Apl I. **A** Twenty-five models overlayed and aligned by the PKM, showing a consistent KIBRA_wt_ substrate (green) path through PKM Apl III (model ipTM range 0.87–0.88, all KIBRA_wt_ structures hidden except the canonical PKM substrate (1310–1323) (left). Half of the models show a similar substrate path through PKM Apl I (green, 13 of 25 models, with ipTM range 0.8–0.86), however the other half of the models with PKM Apl I show an alternate path for this peptide with ipTM range 0.65–0.68 (9 models) or low confident paths < 0.6 ipTM (3 models) (orange), often interacting with the handle region (olive) of PKM Apl I. Sequence alignment of the Human Par3 substrate and the Aplysia KIBRA substrate residues consistently modelled to be involved in interactions with PKM Apl III numbered relative to the potential phosphorylation residue, P-0 marked as *. **B** (i). Human PAR3 (blue) and Aplysia KIBRA_WT_ (green) substrate ‘affinity arm’ side chains of -9 F in the hydrophobic pocket (Ψ1), and − 7R and − 2R in separate acidic pockets of PKM Apl III (PKM surface in grey/yellow, acidic side chains of PKM Apl III in red, hydrophobic side chains of PKM Apl III in white). (ii) The substrate modeled with PKM Apl I, has a similar path (ii, green) or an alternative path (iii, orange) involving − 9 F interacting with the hydrophobic pocket in the handle (Ψ2) while − 7R interacts with the same pocket as -2R. D192 in PKM Apl I permits this alternative interaction with both − 7R and − 2R, while the orthologous N185 in PKM Apl III supports only the − 2R with a -3 S hydrogen bond (yellow in Bi). **C** (i) Substrates interacting at the target serine (S) with PKM catalytic loop residues D145/K147, and the + 1 hydrophobic residues sit in a hydrophobic pocket and H-bonds to G182 (PKM side chains predicted to form hydrogen bonds in yellow, hydrophobic interactions in white, ATP in violet, numbering corresponds to PKM Apl III). C(ii) Substrates ‘inhibitory arm’, interacting with three regions in PKM Apl III at Y32&W65 (magenta), at pT179 & F180 (hotpink, white), and at D59 & D62 (red), KIBRA substrate residues +2R, +3 W and +4 K labeled. **D** (i) Par3 interactions with PKM Apl III with residues orthologous to those determined for PKC iota and PAR3 [38], +4 K interacting with pT179, +5R with Y32&W65, and +6T with D62. (ii) KIBRA +2R models suggest a cation-pi interaction to W65 and a salt bridge to pT179, +3 W interacts with the + 1 hydrophobic pocket, and +4 K with the acidic patch at the start of the αC helix, D62 and D59. (iii) The KIBRA substrate inhibitory arm interactions in models with PKM Apl I have a similar +3 W hydrophobic interaction, while +2R is predicted to interact with the primed residue pT186 and D69, while +4 K interacts with D67 and D68 in the αC helix. Representative single models selected based on the absence of severe clashes (> 0.9Å) and Ramachandran plot outliers, and with the lowest mean clash per residue at the substrate interface (KIBRA_WT_ 1310–1323, Human Par3 758–773) as measured with Molprobity [47]; the selected models had the following for substrate interface mean clash per residue, substrate interface pLDDT, and ipTM for Human Par3 in PKM Apl III 0.107Å, 79.69, 0.88. KIBRA_WT_ in PKM Apl III 0.092Å, 73.54, 0.88. KIBRA_WT_ with PKM Apl I green path 0.267Å, 73.06, 0.85. KIBRA_WT_ with PKM Apl I orange alt path 0.434Å, 73.06, 0.65

To gain more insight into how KIBRA and PKM associate and why association may not lead to stabilization, we used AlphaFold 3 [1] to examine the predicted interaction between forms of KIBRA (KIBRA_WT_, KIBRA_splice_, KIBRA_RSR−AAA_) and PKM Apl III. We also examined the predicted interaction of these forms of KIBRA with PKM Apl I since both KIBRA_splice_ and KIBRA_RSR−AAA_, but not KIBRA_WT_ stabilize PKM Apl I [10]. The most confident interaction predicted by AlphaFold 3 for all KIBRA-PKM interactions is a substrate-like interaction where a PKM-interacting peptide from KIBRA binds in the substrate binding site of PKMs (Fig. 3A). For PKM Apl I, AlphaFold 3 has two possible paths for this peptide, equally represented in the AlphaFold 3 models (Fig. 3A). One path was similar to the one modelled for PKM Apl III and the other is discussed below. The AlphaFold 3 predicted interaction path for the KIBRA substrate peptide with PKM Apl III is very similar to the crystal structure made previously examining the interaction between a peptide sequence in PAR3, that is similar to the KIBRA peptide, and atypical PKC iota [38]. For the PAR3 peptide, interaction sequences surrounding the phosphorylatable serine in the PAR3 peptide leads to inhibition of phosphorylation by PKC iota [38]. These sequences comprise an affinity arm (phenylalanine, X, arginine; FxR) on the N-terminal side of the substrate serine residue and an inhibitory interaction on the C-terminal side of the substrate residue [38]. KIBRA also has the FxR sequence on the N-terminal affinity arm and homology in the C-terminal inhibitory region (Fig. 3A). The AlphaFold 3 model for Human PAR3 with PKM Apl III predicts a similar interaction for PAR3 as seen with PKC iota [38]. In particular, the affinity arm FxR is predicted to have very similar paths with the same interchain interaction residues predicted for PAR3 and KIBRA (Fig. 3B).

KIBRA stabilizes PKM Apl III, but not PKM Apl I, but AlphaFold 3 predicts that the KIBRA peptide can fit into the substrate binding site of both PKMs (Fig. 3Bii). However, there is an alternative predicted binding of PKM Apl I (Fig. 3Biii) where the FxR sequence of KIBRA may interact with what we have termed the handle region of PKM Apl I, a region including an α-helix (helix 5 or helix G in the catalytic domain structure of PKCs) that is important for differential stabilization of PKM Apl I and PKM Apl III [10]. The mean ipTM, an AlphaFold generated metric of interface confidence, is reduced for this alternate substrate path from 0.88 ± 0.01, 0.85 ± 0.01 for KIBRA_WT_ with PKM Apl III and PKM Apl I respectively, to 0.64 ± 0.09 for the alternate substrate path in KIBRA_WT_ and PKM Apl I models (means ± SD from 25 models for PKM Apl III and 13 models for Par-3 like path, 12 models for alternate path for PKM Apl I). The alternate substrate path was modelled with AlphaFold runs over all five models and with a mix of the Par-3 like path and the alternate path, indicating that modelling of the alternate substate path is not solely determined by the initial multiple sequence alignments, the start point of model generation, or the recycling process.

The phosphorylatable serine and + 1 hydrophobic residue are modelled in similar interactions for both PAR3 and KIBRA_WT_ with PKM Apl III and with the corresponding residues of PKM Apl I with KIBRA_WT_ (Fig. 3Ci). However, while the AlphaFold 3 prediction of the interactions of the inhibitory arm of the PAR3 peptide with PKM Apl III are similar to what was predicted for PKM iota (Fig. 3Di), the predicted interactions for the KIBRA peptide with PKM Apl III and for PKM Apl I show differences (Fig. 3Dii and  Diii).

**Fig. 4:**
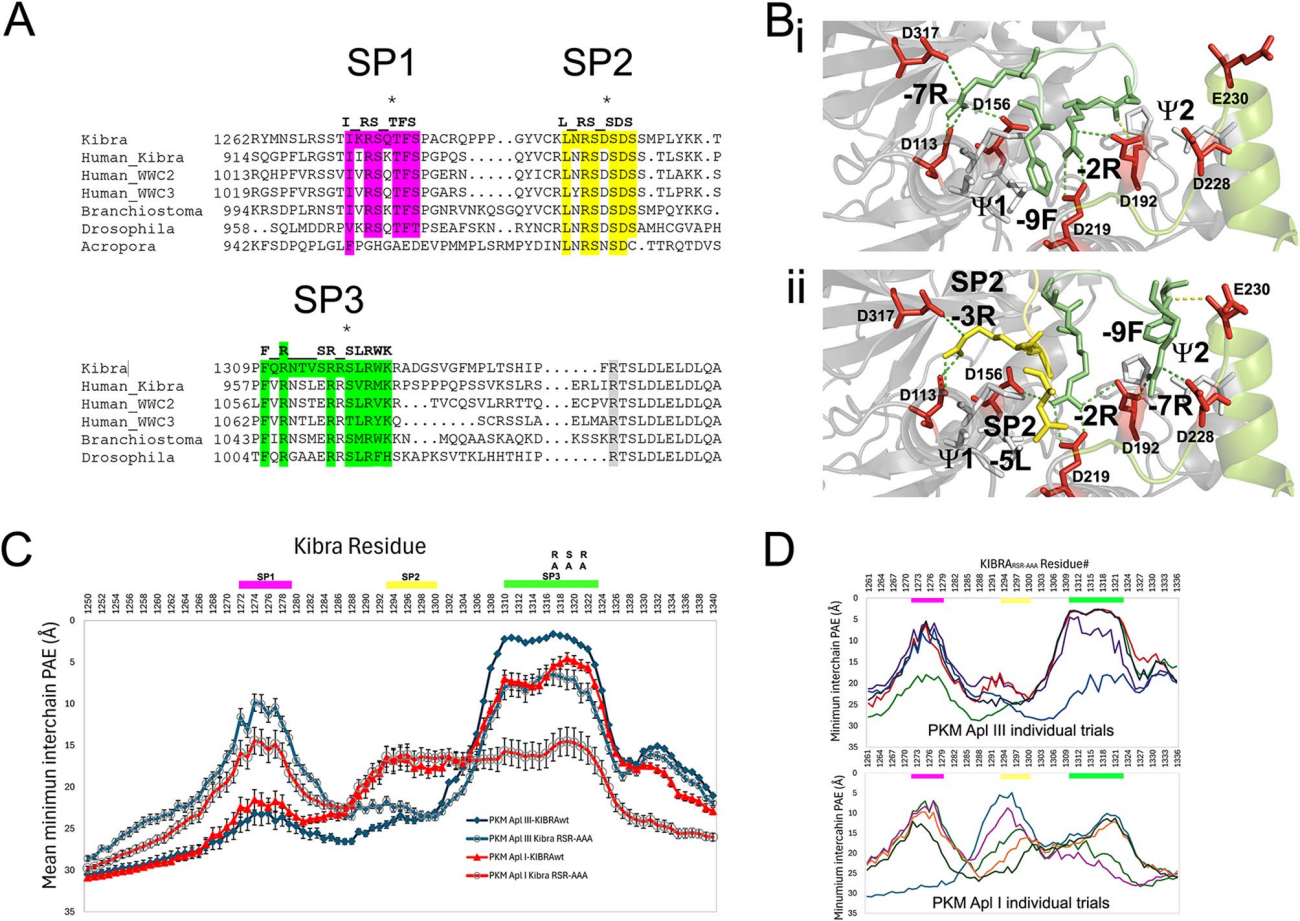
Substrate peptide sequence interactions with PKM Apl III and PKM Apl I. **A** Sequence alignments of KIBRA molecules from Aplysia, Branchiostoma, Drosophila, Acropora, and the three Human isoforms. Bold letters above alignments show residues that can participate in substrate binding in AlphaFold models, with the canonical substrate in green (SP3) and the alternate substrate peptides in magenta (SP1) and yellow (SP2). The R-Q mutation and the KIBRAstop site, R1312 marked in green, and the R-H mutation, R1341 in grey. **B** Representative models of KIBRA_WT_ and PKM Apl I interaction from Fig. 3 with alternate substrate peptides in the − 7R acidic pocket with − 7R alternate binding to the − 2R pocket. Bi) -7R and − 2R of the KIBRA substrate (SP3) with PKM Apl I can interact with similar residues as PKM Apl III, with − 7R and − 2R forming salt bridges in separate pockets of acidic side chains (red). (ii) Alternative binding of -7R KIBRA SP3 peptide (green) to the PKM Apl I handle through D192& D228 allows alternate substrate SP1 or SP2 to interact with the former − 7R pocket formed by D113, D156, D317 (example is of SP2 -3R in yellow). The − 9 F of SP3 is predicted to interact with an alternative hydrophobic pocket (Ψ2) in the PKM Apl I handle region and the − 5 hydrophobic residue of either SP1 or SP2 interacts with the former − 9 F hydrophobic pocket (Ψ1,-5 L of SP2 shown here). Of the 25 models, 12 have the SP3 substrate in the alternate path, involving SP2 (9) or SP1 (3). The model ipTM is reduced from a mean of 0.85 (range 0.8–0.86) for twelve models with the canonical path, to 0.64 for alternate paths (range 0.47–0.68). Acidic sidechains in red, hydrophobic in white. **C** Mean minimum interchain PAE for the substrate binding region of KIBRA for PKM Apl III or PKM Apl I modeled with either KIBRA_WT_ or KIBRA_RSR−AAA_. **D** Minimum interchain PAE of single models with KIBRA_RSR−AAA_ with PKM Apl III (top) or PKM Apl I (bottom). Individual models chosen to represent the variation in model minimum iPAE variation for the substrate region over the twenty-five models to represent the full data set and reduce clutter

AlphaFold 3 also predicts substrate-like binding of alternative peptides. These two alternative sequences, which we call substrate peptide 1 (SP1) and substrate peptide 2 (SP2) directly precede the previously identified atypical PKC binding site that we now call substrate peptide 3 (SP3) (Fig. 4A). These regions are highly conserved in KIBRA ([10] Fig. 4A), but the AlphaFold 3 prediction for all substrate peptides is unstructured in KIBRA alone (mean of five models of KIBRA_WT_ alone SP1 to SP3 (aa1271-1323) pLDDT = 35.92. Interestingly, in the alternative path for the KIBRA peptide in PKM Apl I where the FxR may interact with the handle domain, the residues in the kinase domain that were predicted to interact with FxR are now predicted to interact with the comparable regions of SP1 or SP2 (Fig. 4B). To compare these predicted interactions we used the minimum interchain predicted alignment error (iPAE) calculation from AlphaFold 3 that measures the confidence in residue proximity to all other residues. We use the mean of 25 models (see Methods) to calculate the mean minimum iPAE for each residue which gives a measure of potential interaction [1]. PKM Apl III had lower iPAE (higher predicted interaction stability) in the SP3 region than PKM Apl I and higher iPAE (lower predicted interaction stability) with SP1 and SP2 than PKM Apl I (Fig. 4C). These potential interactions change with KIBRA_RSR−AAA_. This dramatically shifts the predicted PKM interactions to SP1 from SP3 for both PKM Apl I and PKM Apl III (Fig. 4C). While the mean iPAE is useful, it can be misleading as it averages across quite distinct models. To illustrate this point, we plot 5 individual iPAE calculations in Fig. 4E to show this variability. Thus, while some models still show strong predicted interaction of SP3 even in the absence of the conserved RSR sequence, a substantial number of models show higher iPAE for SP3 and lower iPAE for either SP1 or SP2 lowering the mean iPAE of SP3 (Fig. 4D). The ability of the other substrate peptides to replace SP3 may explain why PKM Apl III still shows BIFC with KIBRA_RSR−AAA_.

We used Molprobity [47] to choose the best model with the least molecular clashes (see Methods). It should be noted that the number of clashes is relatively high for the best models of PKM Apl I and KIBRA_WT_ (Fig. 3; green path 0.267Å,. orange alt path 0.434Å). The best model for PKM Apl I and KIBRA_RSR−AAA_ when SP3 is in the substrate site had fewer clashes (0.208Å; pLDDT = 66.8, iTM = 0.76) as did the best model when SP1 is in the substrate site (0.153 Å; pLDDT = 58.2, ipTM = 0.6). In contrast, Molprobity shows no strong model (mean clash pre residue < 0.9 A and 100% favored Ramachandran scores) for PKM Apl III and KIBRA_RSR−AAA_ with SP3 in the binding pocket and the number of clashes is high with SP1 (0.414 Å; pLDDT = 53.1, ipTM = 0.71).

### A positively charged alpha-helix in KIBRA that interacts with different regions of PKM Apl I and PKM Apl III gains negative changes after splicing

**Fig. 5:**
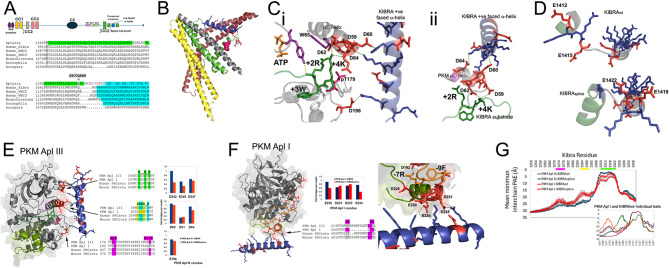
Splice insert differentially alters interaction of positively charged α-helix with PKM Apl III and PKM Apl I. **A** Domain map of *Aplysia* KIBRA and amino acid sequence alignment of KIBRA from *Aplysia*, *Branchiostoma*, *Drosophila*, and *Acropora*, and the three Human orthologs, KIBRA, WWC2, and WWC3. The R-H mutation is marked as are the locations of three conserved post-PKC substrate helices, the first is the other half of the second coiled coil domain (green in B), the second a-helix has the splice site marked in grey with the insert noted above, and the third α-helix highlighted in cyan has a face of positive charged side chains R, K,H, in blue in Aplysia. The two residues with acidic side chains that enter the positive face of the helix with splice in red. **B** Representative cartoon structure generated by AlphaFold 3 of Aplysia KIBRA colored according to A, with CC1 in yellow, CC3 in red, CC2 grey/green. Only CC regions and post-substrate alpha helices are visible with the post-substrate helices in green and the positive faced helix in blue with the splice site marked with pink spheres. Residue side chains visible and colored according to charge for K, R,H, E,D from the splice site through the positive faced helix. **C** i. AlphaFold 3 models of the inhibitory arm of KIBRA_WT_ SP3 (+ 2RWK) interacting with PKM Apl III as in Fig. 3Dii, now also showing potential concurrent interactions from the positive faced helix that tends to be modeled salt bridging to the other two aspartic acid residues in the αC helix acidic patch (D60,D64). 22 of 25 models of PKM Apl III with KIBRA_WT_predict an interaction with D60 and D64 with the positive-faced helix. ii) Different viewing angle of i, with just the side chains of the residues participating in salt bridges formed with the αC helix visible. This is an example, specific residues interacting from the positive faced helix vary model to model, reducing the average iPAE of this interaction. **D** The positive faced helix with all charged side chains visible has nine residues with positive charged side chains facing one direction and E1412&E1415 side chains facing away (top). In all 25 models, including the 16 models without Ramachandran outliers in this region, E1412& E1415 are modelled to face away from the positive faced helix in KIBRA_WT_. The splice site with the 7 amino acid insert (green) in KIBRA_splice,_ the negative charged E1412&E1415, now E1419&E1422 are predicted by AlphaFold to participate in the helix with the negative side chains in the same direction as the positive face of the helix (bottom). This modelling of E1419& E1422 into the positive face in KIBRA_splice_ occurs in 11 of 14 models without Ramachandran outliers over this region. **E** Representative Alphafold 3 generated model of PKM Apl III with KIBRA, only SP3 (green) and the positive faced helix visible from KIBRA (K, R,H and D, E side chain sticks visible, blue and red respectively). The positive faced helix interacts with three regions in PKM Apl III, D342,D345,D347 (green), E196, G178, pT179 (magenta), and D60,E61,D64 in the negative charged patch in inhibitory arm interacting region (cyan). Sequence alignments highlight the similarities between atypical PKC/PKM and PKM Apl I, coloring shows conservation, with grey highlighting differences in PKM Apl I and yellow showing the residues interacting the KIBRA substate at the inhibitory arm interacting region. **F** Representative model of the alternative interaction with PKM Apl I and KIBRA_WT_, only SP3 (orange) and the positive faced helix visible from KIBRA. The positive faced helix interacts with a single region in the handle of PKM Apl I (yellow) at E230,D231,E233,E234 (red), where the interactions with E230 and E234 decrease in KIBRA_splice_ models. (Charts in E&F) Mean predicted number of salt bridges formed between the KIBRA positive faced α-helix and specific residues in the PKMs over twenty-five models for each KIBRA_WT_ and KIBRA_splice,_ normalized per model. With PKM Apl III, the positive faced helix interacts consistently with three locations, two of which have a reduced number of interactions when modelled with KIBRA_splice_. With PKM Apl I the positive faced helix interacts with a single region in the handle and this interaction is reduced with two of the residues E230 and D234 in KIBRA_splice_ models, compared to KIBRA_wt_. Right, closeup of the patch of acidic side chains (red) in the PKM Apl I handle (olive) that participate in the alternate SP3 binding (-7R orange) and interaction with the positive faced helix of KIBRA_WT_ (grey, with positive charged side chains in blue). **G** Mean minimum iPAE for the KIBRA substrate region of models with KIBRA and KIBRA_splice_ with either PKM Apl III or PKM Apl I. Representative individual models in inset show variability in minimum iPAE between models with PKM Apl I and KIBRA that lead to increased mean minimum iPAE at SP1 and SP2. Specific representative models are the same used in Fig. 3 and the model for KIBRA_splice_ with PKM Apl III for 5D had a substrate interface mean clash per residue 0.035Å, substrate interface pLDDT = 73.15, and ipTM = 0.87

KIBRA_splice_ stabilizes PKM Apl I, but not PKM Apl III, but KIBRA_splice_ shows strong BIFC with PKM Apl III. The splice occurs in a region of KIBRA after the SPs that AlphaFold 3 predicts consists of four alpha helices (Fig. 5A). The first of the helices is predicted to form the second coiled-coil (CC) domain with one of the helices from the N-terminal region. The splice occurs between the second and third predicted alpha helices. All the sequences predicted to form these helices are highly conserved [10]( Fig. 5A), and either are predicted to be part of or sandwiched in between the three CC domains (Fig. 5B). These interactions and helices are also predicted for vertebrate KIBRA (WWC1), WWC2 and WWC3 (Supplementary Fig. 1). In particular, the third of these helices is predicted to have a face with mainly positive charged amino acid side chains. Some of these positively charged residues are predicted to interact with the region of PKM Apl III predicted to interact with the inhibitory arm of the KIBRA substrate peptide (Fig. 5C). The splice added seven amino acids to the linker between two of these helices (Fig. 5B) and importantly, is predicted to allow negatively charged residues previously in the linker region between the second and third predicted alpha-helix to join the face of the helix (Fig. 5D). The pLDDT measure of Alphafold3 folding confidence of the splice, linker, and positive faced helix was similar with KIBRA_WT_ 63.38 and KIBRA_splice_ 63.07 (residues 1406–1439 for KIBRA_WT_ and 1406–1446 for KIBRA_splice_), suggesting the difference between structures in not a result of a change in folding confidence but rather a potential real structural change that may underlie the observed differences with KIBRA_splice_. Consistent with this structural change underlying the difference with KIBRA_splice_, the AlphaFold 3 models with KIBRA_splice_ are predicted to have reduced interactions between the positively charged face of the helix and PKM Apl III (Fig. 5E), also apparent as a change in the mean minimum iPAE at the helix (from 14.8 ± 1.0 to 17.6 ± 2.1Å, for PKM Apl III with positive faced helix of KIBRA_WT_ (1419–1440) and KIBRA_splice_ (1419–1446) respectively, mean over 25 models±pooled SD). Some of the negative charges important for the predicted interaction of the positively charged helix with PKM Apl III are not conserved in PKM Apl I (Fig. 5E). In particular, an arginine (gray) at position 185 in PKM Apl I is predicted to strongly decrease the interaction (Fig. 5E). Instead, for PKM Apl I the helix is predicted to interact with a group of negative charged side chains in the handle domain, a region not conserved in PKM Apl III (Fig. 5F). Some of these interactions are modelled to reduce in KIBRA_splice_, particularly with the two acidic residues closest to the alternative − 7R binding pocket (Fig. 5F). The ‘affinity arm’ interacting FxR of KIBRA SP3 is often modelled interacting with the PKM Apl I handle (Fig. 3) while either SP1 or SP2 is predicted to interact with the affinity arm-interacting region of PKM Apl I, something that is not observed in models with PKM Apl III. The occurrence of this predicted alternate interaction of the canonical substrate peptide SP3 with the PKM Apl I handle domain is reduced in models with KIBRA_splice_ (5 of the 25 models, compared to 12 of 25 models with KIBRA_WT_). The AlphaFold models of KIBRA_splice_ with PKM Apl I also have reduced interactions with the positively charged alpha helix and the handle domain, particularly with the two acidic residues E230 and E234 (Fig. 5F inset chart), the closest acidic residues to D228 which is modelled to be a key interaction supporting the alternate − 7R binding site in the − 2R pocket of PKM Apl I (Fig. 5Ffar right). The mean minimum iPAE of PKM Apl I for SP3 is decreased (predicted interaction increased) with KIBRA_splice_ as it is predicted to be more likely that KIBRA SP3 follows the path that is similar to that predicted of PKM Apl III (Fig. 5G). This is correlated with increased stabilization of PKM Apl I by KIBRA_splice_ [10].

### Effects of KIBRA variants on stabilization and BIFC

Several mutations in WWC3 in the substrate binding and helices region cause neurodevelopmental disorders in humans or transmissible cancer in vertebrates [18, 19, 40]. To determine the importance of these mutations we converted the orthologous residues in *Apysia* KIBRA and KIBRA_splice_ to the same amino acid found in these cases (arginine at residue 1312 to glutamine; KIBRA_R−Q_ and arginine at residue 1341 to histidine KIBRA_R−H_, arginine at residue 1312 to a stop codon KIBRA_stop_; Figs. 4A and 5A). R1312 is the R in FxR affinity arm and R1341 is the first residue in the first alpha helix that participates in the CC domains with the N-terminal region of KIBRA (Fig. 5B).

**Fig. 6:**
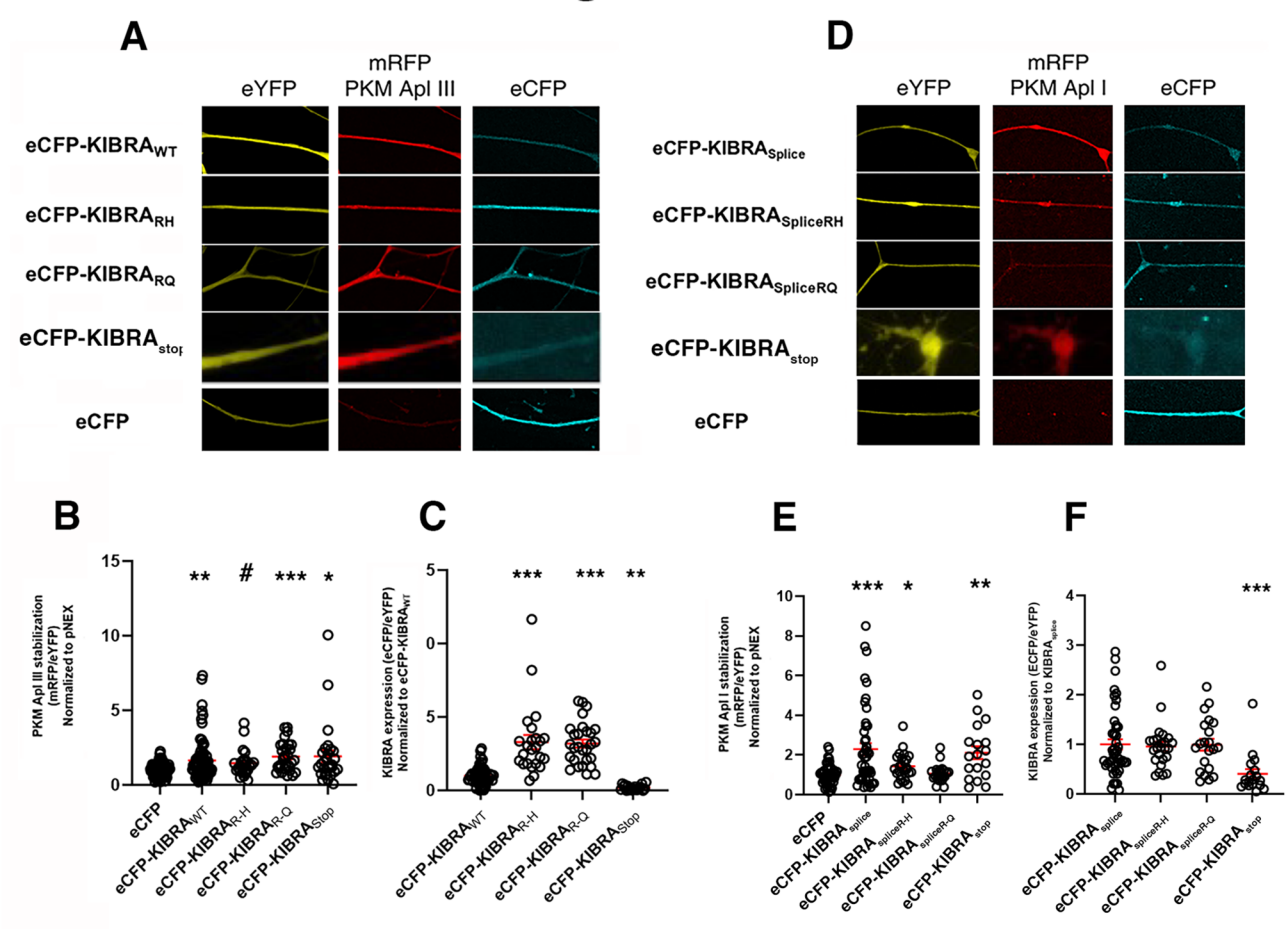
Stabilization of KIBRA variants. **A** Representative examples of expression of eYFP (control for injection) eCFP (eCFP or eCFP-tagged KIBRA) and mRFP (PKM Apl III) for the five groups (eCFP, eCFP-KIBRA_WT_, eCFP-KIBRA_R−H_, eCFP-KIBRA_R−Q_, eCFP KIBRA_stop_ are shown **B** All individual points (black circles) and the average and SEM (red) of the normalized mRFP/eYFP ratio (Stabilization, see Methods) is shown. Kruskal-Wallis test (253,5) = 26.28, *p* < 0.0001. Dunn’s multiple comparison test showed that three of the groups were significantly different than eCFP (*p* < 0.001, ***; *p* < 0.01, **, *p* < 0.05,*). The eCFP-KIBRA_R−H_ group was only of borderline significance (#, *p* = 0.08). The N was the number of sensory neurons quantified (eCFP 89, eCFP-KIBRA_WT_ 84, eCFP-KIBRA_R−H_, 24, eCFP-KIBRA_R−Q_ 28, eCFP-KIBRA_stop_ 28) from 10 independent experiments. **C** All individual points (black circles) and the average and SEM (red) of the normalized eCFP/eYFP ratio (Expression, see methods) is shown. Kruskal-Wallis test (125,4) = 85.83, *p* < 0.0001. Dunn’s multiple comparison test showed that all of the groups were significantly different than eCFP-KIBRA_WT_ (*p* < 0.001, ***; *p* < 0.01, **). The N and independent experiments is the same as for (B). **D** Representative examples of expression of eYFP (control for injection) eCFP (eCFP or eCFP-tagged KIBRA) and mRFP (PKM Apl I) for the five groups (eCFP, eCFP-KIBRA_splice_, eCFP-KIBRA_spliceR−H_, eCFP-KIBRA_spliceR−Q_, eCFP-KIBRA_stop_ are shown. **E** All individual points (black circles) and the average and SEM (red) of the normalized mRFP/eYFP ratio is shown. Kruskal Wallis test (159,5) = 21.84, *p* < 0.001. Dunn’s multiple comparison tests showed that eCFP-KIBRA_splice_ (*p* < 0.001, ***), eCFP-KIBRA_spliceR−H_ (*, *p* < 0.05) and eCFP-KIBRA_stop_ (**, *p* < 0.01) were significantly different that eCFP. In contrast, eCFP-KIBRA_spliceR−Q_ was not significantly different that eCFP (*p* > 0.5). The N was the number of sensory neurons quantified (eCFP 48, eCFP-KIBRA_splice_ 46, eCFP-KIBRA_spliceR−H_ 25, eCFP-KIBRA_spliceR−Q_ 22, eCFP-KIBRA_stop_ 18) from 8 independent experiments. **F** All individual points (black circles) and the average and SEM (red) of the normalized eCFP/eYFP ratio (Expression, see methods) is shown. Kruskal-Wallis test (110,4) = 22.25, *p* < 0.0001. Dunn’s multiple comparison test showed that only eCFP-KIBRA_stop_ was significantly different than eCFP-KIBRA_WT_ (*p* < 0.001, ***; The N and independent experiments is the same as for E

For stabilization assays, KIBRAs were generated as fusion proteins with eCFP at the N-terminus to allow comparison of expression levels. The level of mRFP-PKM Apl III was increased by eCFP-KIBRA_WT_, eCFP- KIBRA_R−H_ and eCFP-KIBRA_R−Q_ when standardized to the eCFP alone group (Fig. 6A; quantified in Fig. 6B). Surprisingly, expression of CFP-KIBRA_stop_ in which the proposed PKM binding site and the helices that contribute to the coiled-coil region in the C-terminal is removed still stabilized PKM Apl III (Fig. 6A; quantified in Fig. 6B). All these mutants were expressed at higher levels than CFP-KIBRA_WT_ (Fig. 6C), except for CFP-KIBRA_stop_ that was expressed at significantly lower levels (Fig. 6C). For PKM Apl I stabilization all mutations were made in the CFP-KIBRA_splice_ background as PKM Apl I is stabilized by KIBRA_splice_, but not KIBRA_WT_. The stop codon in KIBRA_stop_ precedes the splice so this construct was the same in both assays. In contrast to PKM Apl III, CFP-KIBRA_spliceR−Q_ did not stabilize PKM Apl I, while CFP-KIBRA_splice_, CFP-KIBRA_spliceR−H_ and CFP-KIBRA_stop_ all stabilized PKM Apl I (Fig. 6D, quantified in Fig. 6E). All CFP-KIBRAs expressed at similar levels, except for CFP-KIBRA_stop_ that was expressed at significantly lower levels (Fig. 6F), consistent with experiment examining stabilization of PKM Apl III (Fig. 6C). AlphaFold3 predictions of the structure of KIBRA_stop_ shows a lack of formation of the coiled coil structures seen in KIBRA_WT_ (Supplemental Fig. 2) and this may lead to decreased stability of this protein.

**Fig. 7:**
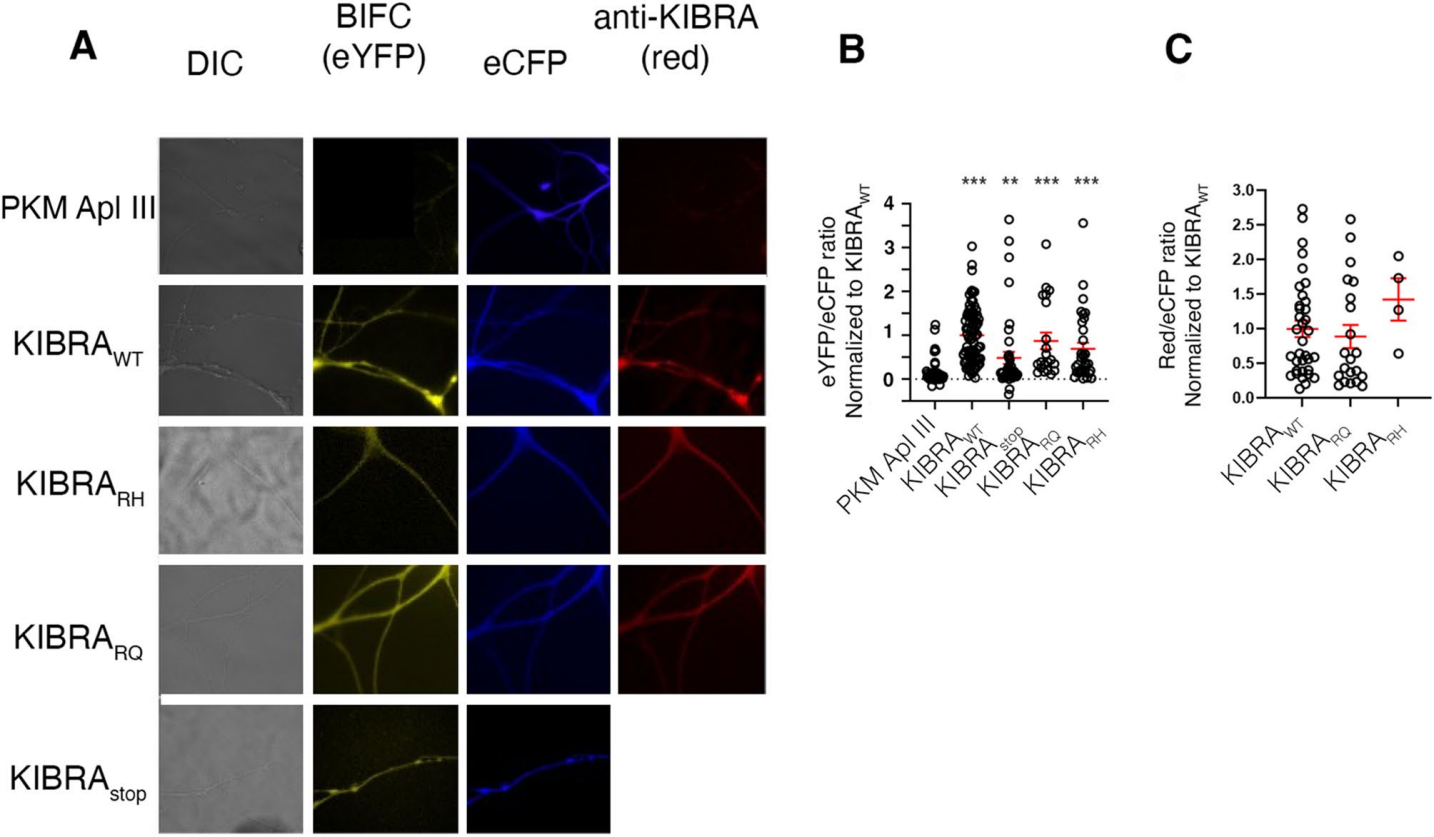
BIFC of KIBRA variants. **A** Representative examples of expression of DIC (Differential Interference Contrast), eYFP (BiFC), Red (Kibra Antibody), and eCFP (control for injection) are seen for the groups all expressing PKM Apl III short and one of: PKM Apl III long, KIBRA_WT_ Long, KIBRA_R−Q_ Long, KIBRA_R−H_ Long, KIBRA_stop_ Long **B** All individual points (black circles) and the average and SEM (red) of the normalized eYFP/eCFP ratio (BIFC, See methods). Kruskal-Wallis test (229,5) = 97.15 showed a significant difference between groups *p* < 0.0001. Dunn’s multiple comparison tests showed that all groups were significantly different that PKM Apl III long (*p* < 0.001, ***, *p* < 0.01, **). N was the number of sensory neurons quantified: PKM Long 52, KIBRA_WT_ Long 82, KIBRA_R−Q_ Long 41, KIBRA_R−H_ Long 33, KIBRA_stop_ Long 41). **C** All individual points (black circles) and the average and SEM (red) of the normalized red/eCFP ratio are shown. Kruskal-Wallis test (72,4) = 31.69 showed a significant difference between groups. Dunn’s multiple comparison tests showed that all groups were significantly different than PKM Long (*p* < 0.001, ***). KIBRA_R−Q_ Long and KIBRA_R−H_ Long were not expressed differently than KIBRA_WT_ Long (*p* > 0.5). N was the number of sensory neurons quantified and different from (B) as not all experiments had antibody staining (PKM Long 12, KIBRA_WT_ Long 35, KIBRA_R−Q_ Long 21, KIBRA_R−H_ Long 4). The antibody does not recognize KIBRA_stop_ as the epitope used to raise the antibody is at the carboxy-terminal

We also compared BIFC levels for KIBRA variants and PKM Apl III. Significant BIFC was seen for all constructs, including KIBRA_stop_ (Fig. 7A, quantified in Fig. 7B). All constructs were expressed at equal levels (Fig. 7C), but since the antibody was generated to a C-terminal peptide, it did not recognize KIBRA_stop_ and we assume that it expressed at lower levels that the other constructs based on levels seen with eCFP-KIBRA_stop_ (Fig. 6).

**Fig. 8:**
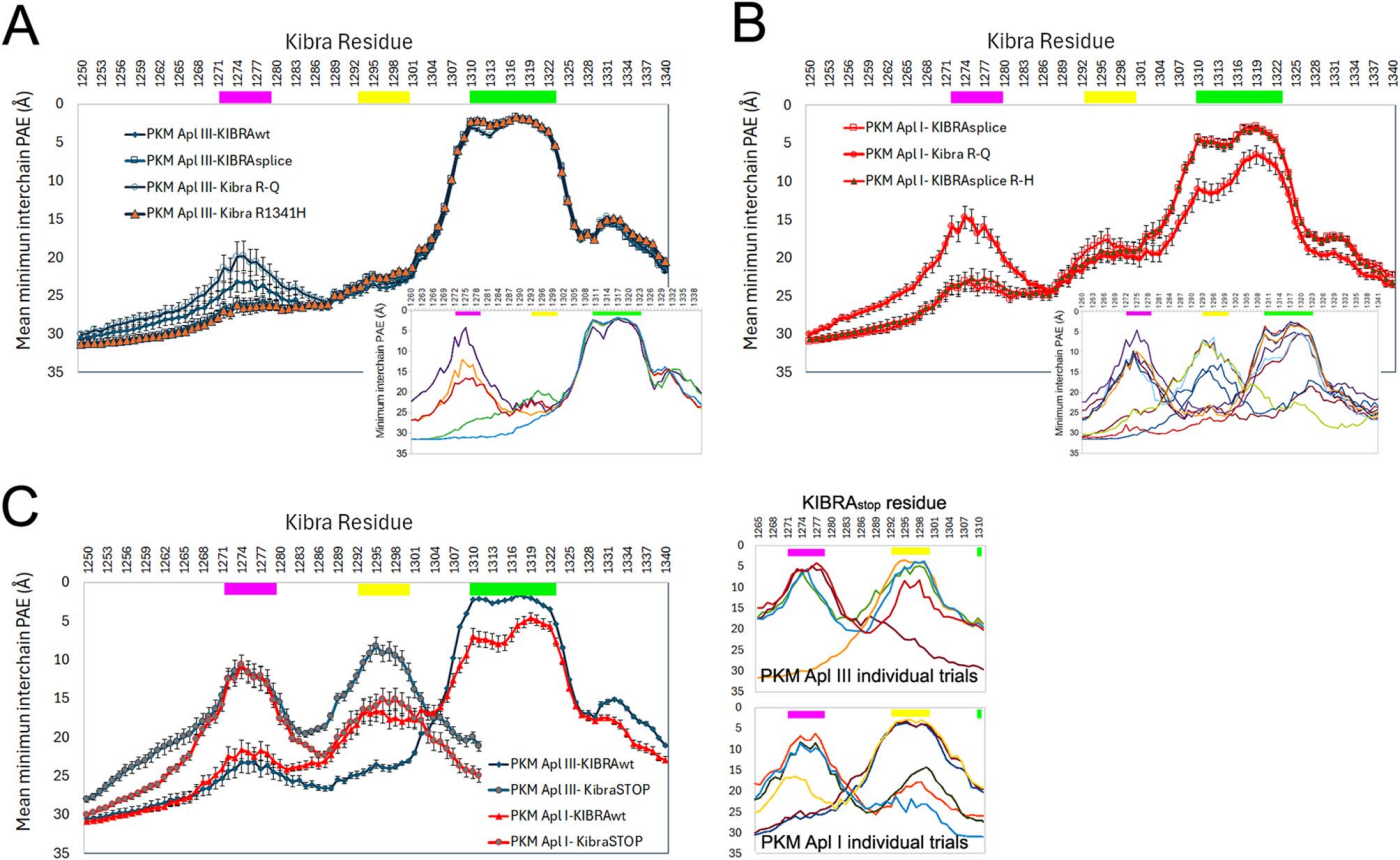
AlphaFold 3 models of KIBRA variants. Mean minimum interchain PAE for KIBRA substrate region for KIBRA mutants, KIBRA R-Q (1312) and KIBRA R-H (1341) with PKM Apl III (**A**) or PKM Apl I (**B**) (measured from 25 models in each group). Insets show representative individual AlphaFold model minimum iPAE measurements revealing variation in interaction sites between individual models. **C** Mean minimum interchain PAE for KIBRA_stop_ with PKM Apl III and PKM Apl I, with representative individual trials on the right.

We examined the predicted effect of these mutations on the AlphaFold 3 models. There were no major changes predicted with the KIBRA_R-Q_ and KIBRA_R-H_ mutants on PKM Apl III (Fig. 8A), although there was a slight decrease in iPAE for SP1. In contrast, KIBRA_spliceR-Q_ had larger predicted changes in the relative binding of the SPs for PKM Apl I (Fig. 8B) consistent with a larger effect on stabilization for this change. As would be expected given the loss of SP3, KIBRA_stop_ is predicted to show enhanced binding of SP1 and SP2 to the PKMs (Fig. 8C). While the iPAE values for SP1 and SP2 for KIBRA_stop_ appear to show a weaker predicted interaction than seen for SP3 for KIBRA, this is somewhat misleading as it reflects models with either SP1 or SP2. When considering individual models, low iPAE values indicative of a strong interaction are modelled with KIBRA_stop_ (25 PKM Apl III models show peak minimum iPAE of 4.6 ± 0.8). PKM Apl III is modelled as slightly more biased to SP2 and PKM Apl I slightly biased to SP1 (Fig. 8C). This is consistent with the prediction that either SP1 or SP2 alone in interaction with PKMs can lead to stabilization. SP1 and SP2 both start with the affinity arm interacting ΨxR, IxR and LxR, and AlphaFold 3 models suggest these residues interact similarly as SP3 with the PKMs (ipTM 0.69 ± 0.05 and 0.64 ± 0.05 for KIBRA_stop_ models with PKM Apl III that have preference to SP1 (10) or SP2 (15), and 0.62 ± 0.02 and 0.74 ± 0.03 for KIBRA_stop_ models with PKM Apl I with preference to SP1 (18) and SP2 (6) with one model not showing a confident substrate interaction). The mean clash per residue is low for the KIBRA_stop_ alternate substrates, as low as 0.189Å and 0.112Å with PKM Apl III and as low as 0.066Å and 0.063Å with PKM Apl I for models preferring SP1 and SP2 respectively (data from models selected with the same criteria as for SP3, no severe clash (> 0.9 A) 100% Ramachandran favored residues). 

## Discussion

Overall, our results highlight the role of predicted substrate-like peptides in KIBRA for Alpha-Fold 3 predicted KIBRA-PKM interactions. The AlphaFold 3 models show differences in this interaction with KIBRA variants, and between PKM Apl I and PKM Apl III. Examining the iPAEs in the SP1, SP2 and SP3 regions together with examining of the clashes predicted by Molprobity for the models [47] provide hypotheses to explain the results we observe. The change in clashes with KIBRA_RSR−AAA_ for PKM Apl I (decreased clashes) to PKM Apl III (increased clashes) is consistent with switches in the stabilization of PKM Apl I and PKM Apl III by KIBRA_RSR−AAA_. Similarly, the decreased clashes in KIBRA_stop_ for both PKM Apl I and PKM Apl III are consistent with the stabilization of both isoforms by this construct. Alpha Fold 3 predictions partially explained the differential effects of the splice on PKM Apl I and PKM Apl III. It predicts the insertion of negative changes into the alpha helix to change its interactions, and since the alpha helix affected by the splice is predicted to interact very differently with PKM Apl I and PKM Apl III suggests reasons the splice affects the two interactions distinctly. However, the reasons for the differential stabilization of PKM Apl I and PKM Apl III by KIBRA_splice_ is not explained by these differences. We suggest that a critical feature that is difficult to predict from the models is whether the substrates will actually be phosphorylated or the binding leads to strong inhibition as experimentally shown for the PAR3 and KIBRA peptides for the atypical PKC iota [38]. Thus, we would predict that KIBRA_splice_ no longer stabilizes PKM Apl III because alterations in the interaction between the inhibitory arm of the KIBRA peptide with PKM Apl III due to splicing leads to increased phosphorylation of the KIBRA peptide. It should be noted that even the PAR3 peptide in isolation shows some phosphorylation by atypical PKCs using more sensitive assays [13]. We would predict the opposite effect for PKM Apl I, stabilization of an inhibitory state after the splice.

The possible role of KIBRA as an inhibitor of PKCs/PKMs is consistent with some models [51], but not others [45, 44]. Like the PAR3 peptide, and unlike a pseudosubstrate, there is a phosphorylatable residue in the KIBRA SPs. In the polarity pathway PAR3 stabilizes atypical PKC in an apical complex where the kinase is inhibited by this peptide. Activation of PKC phosphorylation of PAR3 breaks up the complex leading to PAR3 re-localizing to junctions and PKC phosphorylation of key substrates in organizing cell polarity [38]. Similarly, atypical PKC binds to its substrate LGI in a stalled configuration with binding to cdc-42 activating phosphorylation and dissociation [7]. We would suggest that regulated phosphorylation of SPs in the KIBRA-PKM interaction regulates the function of this complex.

The simplest interpretation of the discrepancies we observe between BIFC and stabilization is to assume that BIFC interactions are more sensitive to transient events than stabilization. This is not surprising, since with some probability transient interactions will lead to joining of the two parts of the fluorescent proteins and once joined, this interaction may no longer require continuous PKM-KIBRA interactions. In contrast, we envision that destabilization of PKMs occur when PKMs are not associated with KIBRA and that leads to degradation of the kinase. Once the protein is degraded, it can no longer be protected by KIBRA. Thus, we expect to see association with BIFC with semi-stable interactions, but not stabilization since semi-stable interactions would allow for degradation. This model is consistent with the data we observe, seeing BIFC without stabilization, but not the reverse.

We had shown previously that isoform specific stabilization of PKMs by KIBRA depended on differences in the “handle” domain [10]. Similar to PKM Apl I, PKM Apl III containing the PKM Apl I handle is stabilized by KIBRA_splice_, but not KIBRA. Moreover, small molecules that bind to the handle domain act as PKC inhibitors and reduce PKMζ BIFC with KIBRA [44]. Thus, we expected that AlphaFold 3 would predict direct interactions between the handle domains and KIBRA. We do observe predicted interactions between the handle domain of PKM Apl I, both with the affinity arm of the substrate peptide and the alpha helix following the splice and these interactions are consistent with the effects of stabilization when the PKM Apl I handle replaces the PKM Apl III handle. In contrast, the PKM Apl III handle domain is predicted to show quite weak interaction with KIBRA and is the only region of PKM Apl III that shows high iPAE in the KIBRA-PKM AlphaFold 3 model (Supplemental Fig. 3). This is conserved in the predicted PKMζ-KIBRA interaction (Supplemental. Figure 3). We have not examined how the atypical PKM inhibitors targeted to the handle domain [44] affect BIFC in *Aplysia* since neither of these inhibitors is expected to bind to PKM Apl III as they are targeted to residues that are not conserved in the *Aplysia* kinase. In a recent paper, the handle domain of PKMζ is predicted to stabilize a hexameric state of PKMζ-KIBRA by interacting between the heterodimers [14]. This region is free in our dimeric structure (Supplementary Fig. 3) and the arginines providing the hydrogen bonds in this model are conserved.

### Limitations of the study

Our results in *Aplysia* sensory neurons are all based on overexpression of KIBRA and PKMs. While this allows for examination of interaction and stabilization in a neuronal context, the level of overexpression is quite high. While we cannot do immunoblots after expression in individual neurons, the level of immunoreactive KIBRA and PKMs compared to non-expressing cells is quite large ([10]Fig. 2). This is important as it is likely that endogenous complexes of KIBRA and PKM contain additional proteins that will affect the interactions we observe but are unlikely to be equimolar with the high concentrations of KIBRA and PKM in these studies. For example, PICK can interact with both KIBRA [29, 37] and PKMs [46, 48]. Similarly, proteins have been identified that bind to the C2, WW and PDZ ligand of KIBRA and these may lead to distinct complexes [12, 35, 41]. Similarly, ligands can bind to the handle domain of PKMs and may compete with KIBRA peptides [27]. PS1 and PS2 have been shown to be RSK substrates [50] and this suggests RSK may be an important regulator of KIBRA-PKM interactions. We believe these additional interactions are necessary for a model where KIBRA-PKM interactions are important for the maintenance of memory, as in this model KIBRA localizes/stabilizes an active form of PKM, while the predicted interactions with KIBRA peptides occupying the substrate binding domain of PKM would act as inhibitors.

## Conclusions

Our experiments demonstrate that BIFC does not predict stabilization for KIBRA-PKM interactions and highlight the importance of KIBRA SPs interacting with PKMs for isolated PKM-KIBRA interactions. We also highlight the possible importance of the highly conserved alpha helices following the KIBRA-PKM interacting peptide in controlling KIBRA-PKM interactions. We would suggest that this inhibitory interaction between KIBRA SPs and PKMs are important at distinguishing synapses not involved in memory and synapses that depend on persistent PKM activity. Activation of phosphorylation of the SPs would free PKM to phosphorylate the substrates important for retaining memory, such as putative endocytic adaptors [9, 31, 34], similar to the role of the PAR3-atypical PKC complex in regulating cell polarity [38]. Understanding the transition from an inhibitory interaction to one where KIBRA complexes with an active PKM is a key question for future studies.

## Supplementary Information

Below is the link to the electronic supplementary material.

Supplementary Material 1

## Data Availability

All custom constructs and antibodies are freely available on request from the Sossin lab. The raw data used and/or analysed during the current study are available from the corresponding author on reasonable request.

## Abbreviations

BIFC: Bimolecular fluorescence complementation
CC: Coiled-coil
CFP: Cyan fluorescent protein
GFP: Green fluorescent protein
iPAE: Interchain predicted alignment error
KIBRA: Kidney brain protein
LATS: Large tumor suppressor kinase
LTF: Long-term facilitation
PAR3: Partitioning defective 3
PKC: Protein kinase C,
PKM: Protein kinase M
mRFP: Monomeric red fluorescent protein
ROI: Region of interest
SP: Substrate peptide
WWC: WW and C2 domain containing
eYFP: Enhanced yellow fluorescent protein

## References

[1] Abramson J, et al. Accurate structure prediction of biomolecular interactions with AlphaFold 3. Nature. 2024;630:493–500. 38718835 10.1038/s41586-024-07487-w PMC11168924

[2] Bates TC, Price JF, Harris SE, Marioni RE, Fowkes FG, Stewart MC, Murray GD, Whalley LJ, Starr JM, Deary IJ. Association of KIBRA and memory. Neurosci Lett. 2009;458:140–3. 19397951 10.1016/j.neulet.2009.04.050

[3] Bougie JK, Cai D, Hastings M, Farah CA, Chen S, Fan X, McCamphill PK, Glanzman DL, Sossin WS. Serotonin-induced cleavage of the atypical protein kinase C Apl III in Aplysia. J Neurosci. 2012;32:14630–40. 23077049 10.1523/JNEUROSCI.3026-11.2012 PMC6621423

[4] Cai D, Pearce K, Chen S, Glanzman DL. Protein kinase M maintains long-term sensitization and long-term facilitation in Aplysia. J Neurosci. 2011;31:6421–31. 21525283 10.1523/JNEUROSCI.4744-10.2011 PMC3102530

[5] Carroll M, Dyer J, Sossin WS. Serotonin increases phosphorylation of synaptic 4EBP through TOR, but eukaryotic initiation factor 4E levels do not limit somatic cap-dependent translation in aplysia neurons. Mol Cell Biol. 2006;26:8586–98. 16982686 10.1128/MCB.00955-06 PMC1636790

[6] Dunham TL, Wilkerson JR, Johnson RC, Huganir RL, Volk LJ. WWC2 modulates GABA(A)-receptor-mediated synaptic transmission, revealing class-specific mechanisms of synapse regulation by WWC family proteins. Cell Rep. 2024;43:114841. 39388350 10.1016/j.celrep.2024.114841 PMC11913214

[7] Earl CP, Cobbaut M, Barros-Carvalho A, Ivanova ME, Briggs DC, Morais-de-Sa E, Parker PJ, McDonald NQ. Capture, mutual inhibition and release mechanism for aPKC-Par6 and its multisite polarity substrate Lgl. Nat Struct Mol Biol. 2025;32:729–39. 39762628 10.1038/s41594-024-01425-0 PMC11996676

[8] Farah CA, Hastings MH, Dunn TW, Gong K, Baker-Andresen D, Sossin WS. A PKM generated by calpain cleavage of a classical PKC is required for activity-dependent intermediate-term facilitation in the presynaptic sensory neuron of Aplysia. Learn Mem. 2017;24:1–13. 27980071 10.1101/lm.043745.116 PMC5159657

[9] Farah CA, Dunn TW, Hastings MH, Ferguson L, Gao C, Gong K, Sossin WS. A role for numb in protein kinase M (PKM)-mediated increase in surface AMPA receptors during facilitation in Aplysia. J Neurochem. 2019;150:366–84. 31254393 10.1111/jnc.14807

[10] Ferguson L, Hu J, Cai D, Chen S, Dunn TW, Pearce K, Glanzman DL, Schacher S, Sossin WS. Isoform Specificity of PKMs during long-term facilitation in Aplysia is mediated through stabilization by KIBRA. J Neurosci. 2019;39:8632–44. 31537706 10.1523/JNEUROSCI.0943-19.2019 PMC6820206

[11] Genevet A, Tapon N. The Hippo pathway and apico-basal cell polarity. Biochem J. 2011;436:213–24. 21568941 10.1042/BJ20110217

[12] Heitz FD, Farinelli M, Mohanna S, Kahn M, Duning K, Frey MC, Pavenstadt H, Mansuy IM. The memory gene KIBRA is a bidirectional regulator of synaptic and structural plasticity in the adult brain. Neurobiol Learn Mem. 2016;135:100–14. 27498008 10.1016/j.nlm.2016.07.028

[13] Holly RW, Prehoda KE. Phosphorylation of Par-3 by atypical protein kinase C and competition between its substrates. Dev Cell. 2019;49:678–9. 31163172 10.1016/j.devcel.2019.05.002

[14] Hsieh C, Cano DA, Tsokas P, Cottrell JE, Fenton AA, Shouval H, Sacktor TC. PKMz-KIBRA interactions, molecular turnover and memory. Mol Brain. 2026;19:18. 10.1186/s13041-026-01284-4. 41814337 10.1186/s13041-026-01284-4 PMC12997682

[15] Hu J, Adler K, Farah CA, Hastings MH, Sossin WS, Schacher S. Cell-specific PKM isoforms contribute to the maintenance of different forms of persistent long-term synaptic plasticity. J Neurosci. 2017a;37:2746–63. 28179558 10.1523/JNEUROSCI.2805-16.2017 PMC5354326

[16] Hu J, Ferguson L, Adler K, Farah CA, Hastings MH, Sossin WS, Schacher S. Selective erasure of distinct forms of long-term synaptic plasticity underlying different forms of memory in the same postsynaptic neuron. Curr Biol. 2017b;27:1888–e18991884. 28648820 10.1016/j.cub.2017.05.081 PMC5546621

[17] Hu JY, Baussi O, Levine A, Chen Y, Schacher S. Persistent long-term synaptic plasticity requires activation of a new signaling pathway by additional stimuli. J Neurosci. 2011;31:8841–50. 21677168 10.1523/JNEUROSCI.1358-11.2011 PMC3152308

[18] Jiang YH, et al. Detection of clinically relevant genetic variants in autism spectrum disorder by whole-genome sequencing. Am J Hum Genet. 2013;93:249–63. 23849776 10.1016/j.ajhg.2013.06.012 PMC3738824

[19] Junior E. functional analysis of the WWC3 protein in organogenesis. In: Faculty of biology: University of Munster; 2021.

[20] Kaang BK. Parameters influencing ectopic gene expression in Aplysia neurons. Neurosci Lett. 1996;221:29–32. 9014173 10.1016/s0304-3940(96)13279-1

[21] Kandel ER. The molecular biology of memory storage: a dialogue between genes and synapses. Science. 2001;294:1030–8. 11691980 10.1126/science.1067020

[22] Kauwe G, Pareja-Navarro KA, Yao L, Chen JH, Wong I, Saloner R, Cifuentes H, Nana AL, Shah S, Li Y, Le D, Spina S, Grinberg LT, Seeley WW, Kramer JH, Sacktor TC, Schilling B, Gan L, Casaletto KB, Tracy TE. KIBRA repairs synaptic plasticity and promotes resilience to tauopathy-related memory loss. JCI. 2024;134(3):e169064. 38299587 10.1172/JCI169064 PMC10836803

[23] Kerppola TK. Complementary methods for studies of protein interactions in living cells. Nat Methods. 2006;3:969–71. 17117150 10.1038/nmeth1206-969 PMC2512260

[24] Kodama Y, Hu CD. An improved bimolecular fluorescence complementation assay with a high signal-to-noise ratio. Biotechniques. 2010;49:793–805. 21091444 10.2144/000113519

[25] Kudla J, Bock R. Lighting the way to protein-protein interactions: recommendations on best practices for bimolecular fluorescence complementation analyses. Plant Cell. 2016;28:1002–8. 27099259 10.1105/tpc.16.00043 PMC4904677

[26] Lai HT, Chiang CM. Bimolecular Fluorescence Complementation (BiFC) assay for direct visualization of protein-protein interaction in vivo. Bio Protoc. 2013;3(20):e935. 27390756 10.21769/bioprotoc.935 PMC4932868

[27] Linch M, Sanz-Garcia M, Soriano E, Zhang Y, Riou P, Rosse C, Cameron A, Knowles P, Purkiss A, Kjaer S, McDonald NQ, Parker PJ. A cancer-associated mutation in atypical protein kinase Ciota occurs in a substrate-specific recruitment motif. Sci Signal. 2013;6:ra82. 24045153 10.1126/scisignal.2004068

[28] Lv J, Liang S, Qin P, Liu X, Ge X, Guo Y, Xia S, Jing W, Lu Y, Zhang T, Li H. WWC1 mutation drives dopamine dysregulation and synaptic imbalance in Tourette’s syndrome. Sci Adv. 2025;11:eadr4588. 40153501 10.1126/sciadv.adr4588 PMC11952098

[29] Makuch L, Volk L, Anggono V, Johnson RC, Yu Y, Duning K, Kremerskothen J, Xia J, Takamiya K, Huganir RL. Regulation of AMPA receptor function by the human memory-associated gene KIBRA. Neuron. 2011;71:1022–9. 21943600 10.1016/j.neuron.2011.08.017 PMC3200575

[30] Mendoza ML, Quigley LD, Dunham T, Volk LJ. KIBRA regulates activity-induced AMPA receptor expression and synaptic plasticity in an age-dependent manner. iScience. 2022;25:105623. 36465112 10.1016/j.isci.2022.105623 PMC9713372

[31] Migues PV, Hardt O, Wu DC, Gamache K, Sacktor TC, Wang YT, Nader K. PKMzeta maintains memories by regulating GluR2-dependent AMPA receptor trafficking. Nat Neurosci. 2010;13:630–4. 20383136 10.1038/nn.2531

[32] Papassotiropoulos A, Stephan DA, Huentelman MJ, Hoerndli FJ, Craig DW, Pearson JV, Huynh KD, Brunner F, Corneveaux J, Osborne D, Wollmer MA, Aerni A, Coluccia D, Hanggi J, Mondadori CR, Buchmann A, Reiman EM, Caselli RJ, Henke K, de Quervain DJ. Common Kibra alleles are associated with human memory performance. Science. 2006;314:475–8. 17053149 10.1126/science.1129837

[33] Pawlowski TL, Huentelman MJ. Identification of a common variant affecting human episodic memory performance using a pooled genome-wide association approach: a case study of disease gene identification. Methods Mol Biol. 2011;700:261–9. 21204039 10.1007/978-1-61737-954-3_17

[34] Sacktor TC, Hell JW. The genetics of PKMzeta and memory maintenance. Sci Signal. 2017;10(505):eaao2327–10. 29138296 10.1126/scisignal.aao2327 PMC6171341

[35] Schneider A, Huentelman MJ, Kremerskothen J, Duning K, Spoelgen R, Nikolich K. KIBRA: A new gateway to learning and memory? Front Aging Neurosci. 2010;2:4. 20552044 10.3389/neuro.24.004.2010 PMC2874402

[36] Schrodinger LLC. The PyMOL Molecular Graphics System, 2015. Version 3.1. In.

[37] Shao X, Volk L. PICK1 links KIBRA and AMPA receptor subunit GluA2 in coiled-coil-driven supramolecular complexes. JBC. 2025;301:108397. 10.1016/j.jbc.2025.108397 PMC12136796 40074086

[38] Soriano EV, Ivanova ME, Fletcher G, Riou P, Knowles PP, Barnouin K, Purkiss A, Kostelecky B, Saiu P, Linch M, Elbediwy A, Kjaer S, O’Reilly N, Snijders AP, Parker PJ, Thompson BJ, McDonald NQ. aPKC inhibition by Par3 CR3 flanking regions controls substrate access and underpins apical-junctional polarization. Dev Cell. 2016;38:384–98. 27554858 10.1016/j.devcel.2016.07.018 PMC4998004

[39] Sossin WS. Memory synapses are defined by distinct molecular complexes: a proposal. Front Synaptic Neurosci. 2018;10:5. 29695960 10.3389/fnsyn.2018.00005 PMC5904272

[40] Stammnitz MR, et al. The origins and vulnerabilities of two transmissible cancers in tasmanian devils. Cancer Cell. 2018;33:607–e619615. 29634948 10.1016/j.ccell.2018.03.013 PMC5896245

[41] Swaroop BS, Kanumuri R, Ezhil I, Naidu Sampangi JK, Kremerskothen J, Rayala SK, Venkatraman G. KIBRA connects Hippo signaling and cancer. Exp Cell Res. 2021;403:112613. 33901448 10.1016/j.yexcr.2021.112613

[42] Tokamov SA, Nouri N, Rich A, Buiter S, Glotzer M, Fehon RG. Apical polarity and actomyosin dynamics control Kibra subcellular localization and function in Drosophila Hippo signaling. Dev Cell. 2023;58:1864–e18791864. 37729921 10.1016/j.devcel.2023.08.029 PMC10591919

[43] Tracy TE, Sohn PD, Minami SS, Wang C, Min SW, Li Y, Zhou Y, Le D, Lo I, Ponnusamy R, Cong X, Schilling B, Ellerby LM, Huganir RL, Gan L. Acetylated tau obstructs KIBRA-mediated signaling in synaptic plasticity and promotes tauopathy-related memory loss. Neuron. 2016;90:245–60. 27041503 10.1016/j.neuron.2016.03.005 PMC4859346

[44] Tsokas P, Hsieh C, Flores-Obando RE, Bernabo M, Tcherepanov A, Hernandez AI, Thomas C, Bergold PJ, Cottrell JE, Kremerskothen J, Shouval HZ, Nader K, Fenton AA, Sacktor TC. KIBRA anchoring the action of PKMzeta maintains the persistence of memory. Sci Adv. 2024;10:eadl0030. 38924398 10.1126/sciadv.adl0030 PMC11204205

[45] Vogt-Eisele A, Kruger C, Duning K, Weber D, Spoelgen R, Pitzer C, Plaas C, Eisenhardt G, Meyer A, Vogt G, Krieger M, Handwerker E, Wennmann DO, Weide T, Skryabin BV, Klugmann M, Pavenstadt H, Huentelmann MJ, Kremerskothen J, Schneider A. KIBRA (KIdney/BRAin protein) regulates learning and memory and stabilizes Protein kinase Mzeta. J Neurochem. 2014;128:686–700. 24117625 10.1111/jnc.12480 PMC3947452

[46] Wan Q, Jiang XY, Negroiu AM, Lu SG, McKay KS, Abrams TW. Protein kinase C acts as a molecular detector of firing patterns to mediate sensory gating in Aplysia. Nat Neurosci. 2012;15:1144–52. 22772333 10.1038/nn.3158 PMC4228944

[47] Williams CJ, Headd JJ, Moriarty NW, Prisant MG, Videau LL, Deis LN, Verma V, Keedy DA, Hintze BJ, Chen VB, Jain S, Lewis SM, Arendall WB 3rd, Snoeyink J, Adams PD, Lovell SC, Richardson JS, Richardson DC. MolProbity: more and better reference data for improved all-atom structure validation. Protein Sci. 2018;27:293–315. 29067766 10.1002/pro.3330 PMC5734394

[48] Wu SL, Staudinger J, Olson EN, Rubin CS. Structure, expression, and properties of an atypical protein kinase C (PKC3) from Caenorhabditis elegans. PKC3 is required for the normal progression of embryogenesis and viability of the organism. JBC. 1998;273:1130–43. 10.1074/jbc.273.2.1130 9422779

[49] Xiao L, Chen Y, Ji M, Dong J. KIBRA regulates Hippo signaling activity via interactions with large tumor suppressor kinases. JBC. 2011;286:7788–96. 10.1074/jbc.M110.173468 PMC3048666 21233212

[50] Yang S, Ji M, Zhang L, Chen Y, Wennmann DO, Kremerskothen J, Dong J. Phosphorylation of KIBRA by the extracellular signal-regulated kinase (ERK)-ribosomal S6 kinase (RSK) cascade modulates cell proliferation and migration. Cell Signal. 2014;26:343–51. 24269383 10.1016/j.cellsig.2013.11.012 PMC3894603

[51] Yoshihama Y, Chida K, Ohno S. The KIBRA-aPKC connection: a potential regulator of membrane trafficking and cell polarity. Commun Integr Biol. 2012;5:146–51. 22808318 10.4161/cib.18849 PMC3376049

[52] Zha C, Sossin WS. The molecular diversity of plasticity mechanisms underlying memory: an evolutionary perspective. J Neurochem. 2022;163:444–60. 36326567 10.1111/jnc.15717

[53] Zhou PJ, Xue W, Peng J, Wang Y, Wei L, Yang Z, Zhu HH, Fang YX, Gao WQ. Elevated expression of Par3 promotes prostate cancer metastasis by forming a Par3/aPKC/KIBRA complex and inactivating the hippo pathway. J Exp Clin Cancer Res. 2017;36:139. 29017577 10.1186/s13046-017-0609-y PMC5633884

