# Supplementary material for: Generating models for isoform-specific PKM-KIBRA interactions with BIFC, stabilization and AlphaFold 3
Source: Mol Brain. 2026 Jun 19;19:48. doi: 10.1186/s13041-026-01321-2 (PMC13282866; doi:10.1186/s13041-026-01321-2)
Supplement: Supplementary file 1 — Supplementary Material 1 [file 13041_2026_1321_MOESM1_ESM.pdf]

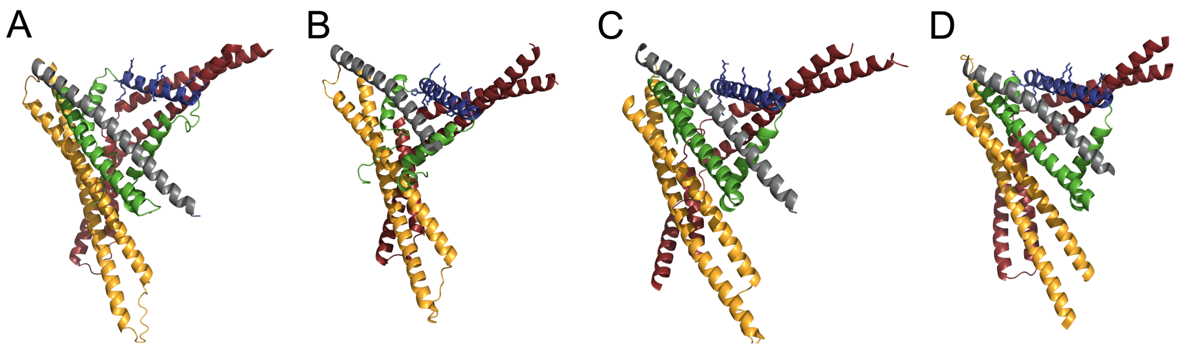

Supplemental Figure 1. Cartoons of representative AlphaFold 3 generated models of *Aplysia* KIBRA, Human KIBRA, WWC2, WWC3 coiled-coil and post-substrate alpha-helices. WW domains, C2 domain, uncertain alpha-helices (removed by aligning five models to CC1 and hiding all regions with greater than 5A difference) and unstructured regions hidden to show alpha-helices participating in the three coiled-coil domains that sandwich three conserved post-substrate binding region alpha-helices. Colored according to 4A, with CC1 in yellow, CC2 in grey/green, CC3 in red, and the post-substrate helices in green and the positive faced helix in blue (sticks of K,R,H side chains visible). Representative models selected with the lowest Molprobity significant clash (>0.4Å) per residue over the CC and post-substrate helices; mean clash per residue and % favored Ramachandran scores for the representative models over these regions are Aplysia KIBRA 0.075Å at 98.8%, Human KIBRA 0.128Å at 99.0%, WWC2 0.099Å at 99.3%, WWC3 0.089Å at 99.0%. Only one run of five AlphaFold models generated for Human KIBRA, WWC2, and WWC3.

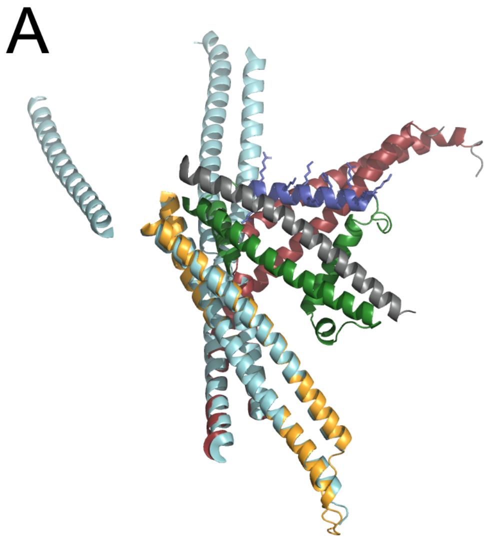

Supplemental Figure 2. Representative AlphaFold generated models of Aplysia KIBRA$_{WT}$ and KIBRA$_{stop}$ with PKM Apl III with only the KIBRA CC domains and post-substrate alpha-helices visible and aligned with first CC alpha-helix pair (CC1, 93-207 in yellow). KIBRA$_{stop}$ in cyan, does not have the post-substrate alpha-helices present in KIBRA$_{WT}$(green, blue), and the CC domains are distorted (CC3, red) or not fully formed (CC2, grey/green).

Representative model for $KIBRA_{WT}$ from supplemental figure 1, the model for $KIBRA_{stop}$ selected as the model with the lowest mean significant clash per residue over the CC regions coded in $KIBRA_{stop}$ (0.061Å with 98.3% favorable Ramachandran scores for $KIBRA_{stop}$ over the CC domains, the $KIBRA_{WT}$ model was 0.063Å and 98.8% over the same regions).

A

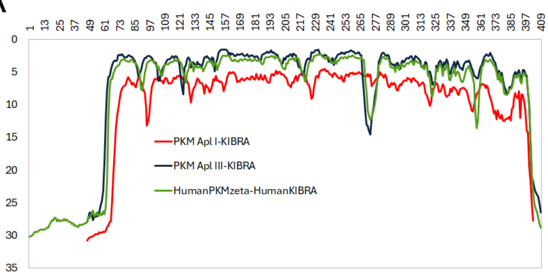

Supplemental Figure 3. Handle domain has lowest iPAE in PKM Apl III. iPAE for PKM Apl I, PKM Apl III and PKMζ with KIBRA. Both PKM Apl III and PKMζ show higher iPAE (lower interaction) in the handle domain compared to PKM Apl I. Note that the sequence numbers are offset for PKM Apl I and PKM Apl III to align with PKMζ. The dip in PKM Apl III representing the handle region is from 221-233 in PKM Apl III, 267-279 in PKMζ.
